# Supplementary material for: Language-specific neural dynamics extend syntax into the time domain
Source: PLoS Biol. 2025 Jan 21;23(1):e3002968. doi: 10.1371/journal.pbio.3002968 (PMC11750093; doi:10.1371/journal.pbio.3002968)
Supplement: S1 Text — (PDF) [file pbio.3002968.s001.pdf]

S1 Text

Coopmans, C. W., de Hoop, H., Tezcan, F., Hagoort, P., & Martin, A. E. Language-specific neural dynamics extend syntax into the time domain.

1. Comparisons against the base model

1.1 Model comparison

S2 Fig A shows the sources in which the reconstruction accuracy of the base model was significantly non-zero. The spatial extent of these clusters is consistent with the location of the auditory cortex, likely reflecting the contributions of the two acoustic predictors in the base model. S2 Figs B-D further show the sources of improvements in reconstruction accuracy when each of the three syntactic predictors are separately added to the base model. For comparison with the results reported in the main manuscript, the scales of the color bars in S2 Figs B-D are identical to those in Fig 4. Spatial cluster-based permutation tests (cluster-level alpha = 0.0083, Bonferroni-corrected for 6 tests) revealed that the top-down predictor explains variance in frontal and temporal regions of the left hemisphere ($t_{av} = 5.08$, $p = .0036$) and to a lesser extent also the right hemisphere ($t_{av} = 3.16$, $p < .001$). The activation pattern for the left-corner predictor is rather similar, with sources of significant explained variance in the left superior temporal lobe ($t_{av} = 4.19$, $p = .0027$) and a spatially more extended area in the right frontal and temporal lobes ($t_{av} = 3.09$, $p < .001$). The bottom-up predictor engages a smaller region centered around Heschl's gyrus, but none of these clusters are significant at the adjusted alpha level ($t_{av} = 3.54$, $p = .0093$).

1.2 Evaluation of the response functions

S2 Fig also shows the TRFs within the significant regions from the cluster-based source analysis of reconstruction accuries, in the left and the right hemisphere separately. The TRF of each syntactic predictor comes from a model which includes all base predictors as well as one syntactic predictor (e.g., the top-

down TRF comes from the Top-down model; see S2 Table). In line with the reconstruction accuracy results, the TRFs show clearly that the neural response to the information encoded in top-down node counts is stronger than the response to node counts derived from a bottom-up or a left-corner parser.

S3 Fig shows the source t-values (based on one-tailed, one-sample t-tests) of the rectified TRFs of the three syntactic predictors. We used spatiotemporal cluster-based permutation tests (cluster-level alpha = 0.0083, Bonferroni-corrected for 6 tests) to determine when and where the TRF coefficients of each syntactic predictor deviated from zero. The results are split up into five time windows (corresponding to different delays in TRF estimation) for the purpose only the figure only.

1.3 Region of interest analysis

The following three regions of interest (ROIs) in the left hemisphere were extracted in the same way as described in the main manuscript: the inferior frontal gyrus (IFG), posterior temporal lobe (PTL) and anterior temporal lobe (ATL; see S4 Fig A). S4 Fig B shows for each ROI the improvement in reconstruction accuracy when the relevant predictor is added to the base model. Like in the main results, two-tailed paired-samples t-tests (with alpha = 0.0056, Bonferroni-corrected for 9 tests) reveal that the addition of the top-down predictor to the base model improves the reconstruction accuracy in all three ROIs (IFG: $t(23) = 4.41$, $p < .001$; PTL: $t(23) = 5.19$, $p < .001$; ATL: $t(23) = 3.55$, $p = .0012$). Adding the left-corner predictor improves reconstruction accuracy in the ATL only, $t(23) = 3.34$, $p = .002$. In each of the three ROIs, we used cluster-based permutation tests to determine when the rectified TRFs of each syntactic predictor differed from each other (at alpha = 0.0056, Bonferroni-corrected for 9 comparisons; 3 TRF pairs * 3 ROIs). The results of this analysis are shown by the horizontal bars below the reponse functions in S4 Fig C.

In all, these results are largely consistent with the results reported in the main manuscript, suggesting that multicollinearity between the predictors did not hinder estimation of the TRF coefficients. However, one notable difference has to do with the left-corner predictor, whose effects are weaker in the results reported in the main manuscript. Regarding the response functions, it is noteworthy that the TRFs of both bottom-up and top-down are stable and quite similar in the full model (Fig 6C in the main

manuscript) and in the simpler models (S4 Fig C), which shows that they are unaffected by the presence of the other syntactic predictors in the full model. The left-corner TRFs, however, are smaller in size and less variable in the full model than in the simpler Left-corner model. In the latter, the left-corner TRFs are stronger than the bottom-up TRFs from the Bottom-up model in an early time window in the PTL and the ATL (S4 Fig C). Moreover, they are more similar to the top-down TRFs from the Top-down model in terms of magnitude and time course, suggesting that these predictors are partially explaining the same variance. In terms of reconstruction accuracy, the explained variance in the Left-corner model attributed to the left-corner predictor (S2 Fig D and S4 Fig B) is visibly reduced when the left-corner predictor is added to a null model that already contains both bottom-up and top-down as predictors (Figs 4C and 6B in the main manuscript). The reverse does not happen, suggesting that some of the variance assigned to the left-corner predictor in the simpler Left-corner model was assigned incorrectly, 'belonging' to top-down rather than left-corner.

2. Correlation between structural and statistical predictors

The correlation matrix in S1 Fig shows that the correlations between word frequency and the three syntactic predictors are quite different. In particular, the correlation between top-down node count and word frequency is effectively zero ($r = -0.081$), while bottom-up node count is positively correlated with word frequency ($r = 0.29$). To determine if this asymmetry can explain the effects we observe, i.e., whether bottom-up explains less variance than top-down because it is more highly correlated with the statistical predictors, we repeated our analysis with TRF models that included no statistical predictors (i.e., word frequency, surprisal and entropy were omitted). Thus, the full model now included the acoustic spectrogram, the acoustic onsets spectrogram, word onset, and the three syntactic predictors. We then determined the unique contribution of each syntactic predictor by comparing the reconstruction accuracy of this full model to the reconstruction accuracy of a null model from which only one of the predictors was omitted. This analysis showed that all syntactic predictors significantly improve reconstruction accuracy (S5 Figs A-C). Spatial cluster-based permutation tests again revealed significant effects in the left hemisphere for all

predictors (top-down: $t_{av} = 6.05$, $p < .001$; bottom-up: $t_{av} = 3.91$, $p = .0082$; left-corner: $t_{av} = 5.31$, $p = .005$). The top-down and left-corner predictors additionally explained variance in the right anterior temporal and inferior frontal lobes (top-down: $t_{av} = 3.40$, $p = .0031$; left-corner: $t_{av} = 3.36$, $p < .001$).

This pattern of results is similar to the results reported in the main manuscript, showing that the relative effects of the syntactic predictors are stable when word frequency, entropy and surprisal are omitted. This means that the correlation between in particular bottom-up node counts and word frequency does not bias the comparison between the three syntactic models against the bottom-up predictor. In fact, if we compare these effects to the effects of the syntactic predictors when the statistical predictors are included (see Fig 4), it becomes clear that removing the statistical predictors leads to the largest increase in reconstruction accuracy for the top-down and left-corner predictors; the effect of the bottom-up predictor remains roughly the same. In other words, the correlation between bottom-up and word frequency, and the inclusion of word frequency as control regressor in our main analysis, does not explain why top-down effects are stronger than the effects of bottom-up node count.

3. Prosodic boundary strength

As shown in S6 Fig D, prosodic boundary strength explained unique variance in frontal regions of the right hemisphere. Importantly, on top of this effect of prosody, the effects of bottom-up (left hem.: $t_{av} = 3.24$, $p = .010$), top-down (left hem.: $t_{av} = 5.01$, $p = .0043$; right hem.: $t_{av} = 2.78$, $p = .035$) and left-corner node counts (left hem.: $t_{av} = 3.41$, $p = .021$; right hem.: $t_{av} = 3.57$, $p = .011$) remained (S6 Figs A-C), albeit reduced in strength. While the top-down effects are quite stable, the effects of bottom-up and left-corner node counts seem to be substantially weaker when we explicitly control for prosodic boundary strength. To test our impression, we compared the increase in reconstruction accuracy for the syntactic predictors when they are added to a full model with boundary strength (i.e., the effects in S6 Fig, discussed here) versus when they are added to a full model without boundary strength (i.e., the effects in Fig 4 of the main manuscript). This analysis yields no difference for the top-down predictor, suggesting that the top-down effect is independent of prosody. In contrast, the variance explained by the bottom-up predictor is significantly reduced by the

addition of prosodic boundary strength as predictor (this reduction centered around left inferior frontal cortex, $t_{av} = 2.71$, $p = .007$), and a similar reduction in explained variance is seen for the left-corner predictor (the cluster centered around left precentral cortex, $t_{av} = 2.22$, $p = .03$). Consistent with the psycholinguistic literature, these results illustrate the neural variance that is shared between prosodic boundary strength and syntactic integration, and point to a potential role for prosody in supporting the inference of syntactic structure.

4. The effect of predictability on integratory structure building

As an exploratory analysis of the interaction between predictability and integratory structure building, we evaluated whether the effect of bottom-up was modulated by the variable surprisal, which reflects the predictability of a word given the preceding context (Slaats et al., 2024; Tezcan et al., 2023). If demands on (syntactic) integration are higher for words that are not predicted, the brain response to bottom-up node counts should be larger for words that are surprising. We computed median surprisal over all stories together and then labeled each word in each story as high surprisal or low surprisal, depending on whether its surprisal was higher or lower than the overall median surprisal. However, splitting the bottom-up predictor in this way leads to two difficulties. First, such a surprisal split is confounded by word duration, because longer words are generally (less frequent and therefore) more surprising (in our stimuli: average length difference = 120 ms, $t(8549) = 37.54$, $p < .001$). We therefore iteratively sampled from the stimuli such that the high- and low-surprisal groups were matched in word length (i.e., almost fully overlapping probability distributions whose means only differed by 0.04 ms, $t(5444) = 0.01$, $p = .99$). The resulting subset contains 64% of the words in original stimulus set. A second difficulty is that splitting the bottom-up predictor by surprisal involves dichotomizing a continuous variable, which is unnatural in both neural terms (i.e., it probably does not represent how the brain transforms this kind of information) and statistical terms (i.e., it reduces power and leads to underestimated effect sizes). As a first check, we therefore compared the reconstruction accuracy of a model in which the bottom-up predictor was split into high- and low-surprisal words to the reconstruction accuracy of an equivalent model in which the bottom-up predictor was split

randomly. This is an unbiased comparison because both models contain a split predictor (Slaats et al., 2024). In the random split, the two groups (bottom-up$_1$ and bottom-up$_2$) were selected randomly but matched in word length (average length difference = 2.03 ms, $t(5444) = -0.47$, $p = .65$). Reconstruction accuracy was significantly larger for the model in which the bottom-up predictor was split by surprisal than for the model in which it was split randomly (largest cluster $t_{av} = 4.01$, $p < .001$). This provides a first indication that splitting bottom-up node counts by their surprisal value captures something relevant; it suggests that predictability modulates the neural response to bottom-up structure building.

To further explore this interaction, we evaluated the effects of bottom-up for high- vs. low-surprisal words. This analysis involved comparing the reconstruction accuracy of a model with bottom-up for only high-surprisal words and a model with bottom-up for only low-surprisal words. Both models contained all other predictors and only differed in whether the bottom-up predictor reflected high- or low-surprisal words. These models did differ in reconstruction accuracy, but not as expected: significantly more variance was explained by the model containing the bottom-up predictor for low-surprisal words than the model containing the bottom-up predictor for high-surprisal words (largest cluster $t_{av} = 3.59$, $p < .001$; S7 Fig A). Likewise, the temporal response function of bottom-up for low-surprisal words is more pronounced (S7 Figs B and C). This suggests, contrary to our hypothesis, that the brain's response to the (syntactic properties of the) bottom-up input is stronger when the input can be predicted (for related results, and a more comprehensive exploration of the relation between syntactic and distributional information, see Slaats et al., 2024).

It thus appears to be the case that predictability modulates integratory structure building, but the story is not as straightforward as sketched in the main manuscript, at least not when a general measure of predictability like surprisal is used. That is, we used *lexical* surprisal (i.e., surprisal about the current word) rather than *structural* surprisal (i.e., surprisal about the syntactic analysis demanded by the current word), which likely affects the interaction with bottom-up integration cost. When structural surprisal is high, the predicted syntactic analysis is wrong, yielding high demands on bottom-up structural integration. In contrast, when lexical surprisal is high, it simply means that the current word was not predicted. But that

does not necessarily mean that syntactic integration is costly, as it might very well be that the word's part of speech was predicted correctly, due to which integrating that (surprising) word into the syntactic structure is relatively straightforward. Another complicating factor is that the surprisal split is confounded not only with word duration, but also with word frequency (because surprisal is correlated with frequency, see S1 Fig) and to a lesser extent also with entropy and the position of a word in a sentence (i.e., words in early-sentence positions are generally more surprising). All of these correlations might affect the pattern we see here, which underscores the importance of incorporating interactive effects in encoding models of language-related brain activity.

References

Slaats, S., Meyer, A. S., & Martin, A. E. (2024). Lexical Surprisal Shapes the Time Course of Syntactic Structure Building. *Neurobiology of Language*, *5*(4), 942–980. https://doi.org/10.1162/nol_a_00155

Tezcan, F., Weissbart, H., & Martin, A. E. (2023). A tradeoff between acoustic and linguistic feature encoding in spoken language comprehension. *eLife*, *12*, e82386. https://doi.org/10.7554/eLife.82386
