# Supplementary figures and images for: Language-specific neural dynamics extend syntax into the time domain
Source: PLoS Biol. 2025 Jan 21;23(1):e3002968. doi: 10.1371/journal.pbio.3002968 (PMC11750093; doi:10.1371/journal.pbio.3002968)

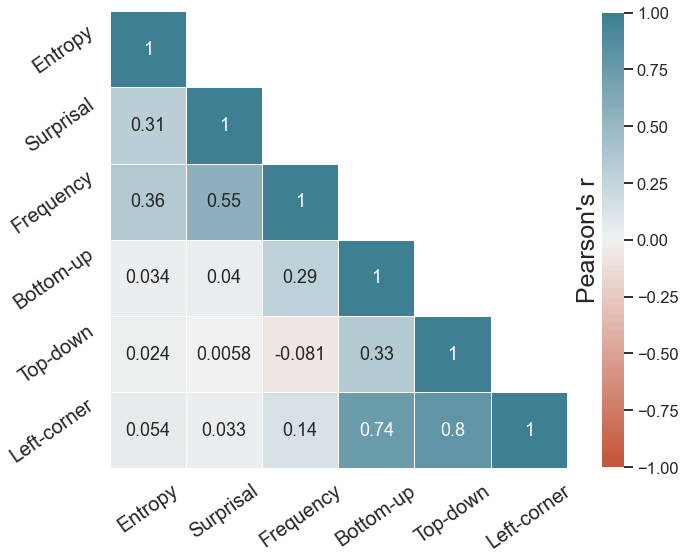

Supplement: S1 Fig — The values correspond to the Pearson correlation for each pair of predictors. Data are available on the Radboud Data Repository (https://doi.org/10.34973/m1vp-hc15). (TIFF) [file pbio.3002968.s002.tiff]

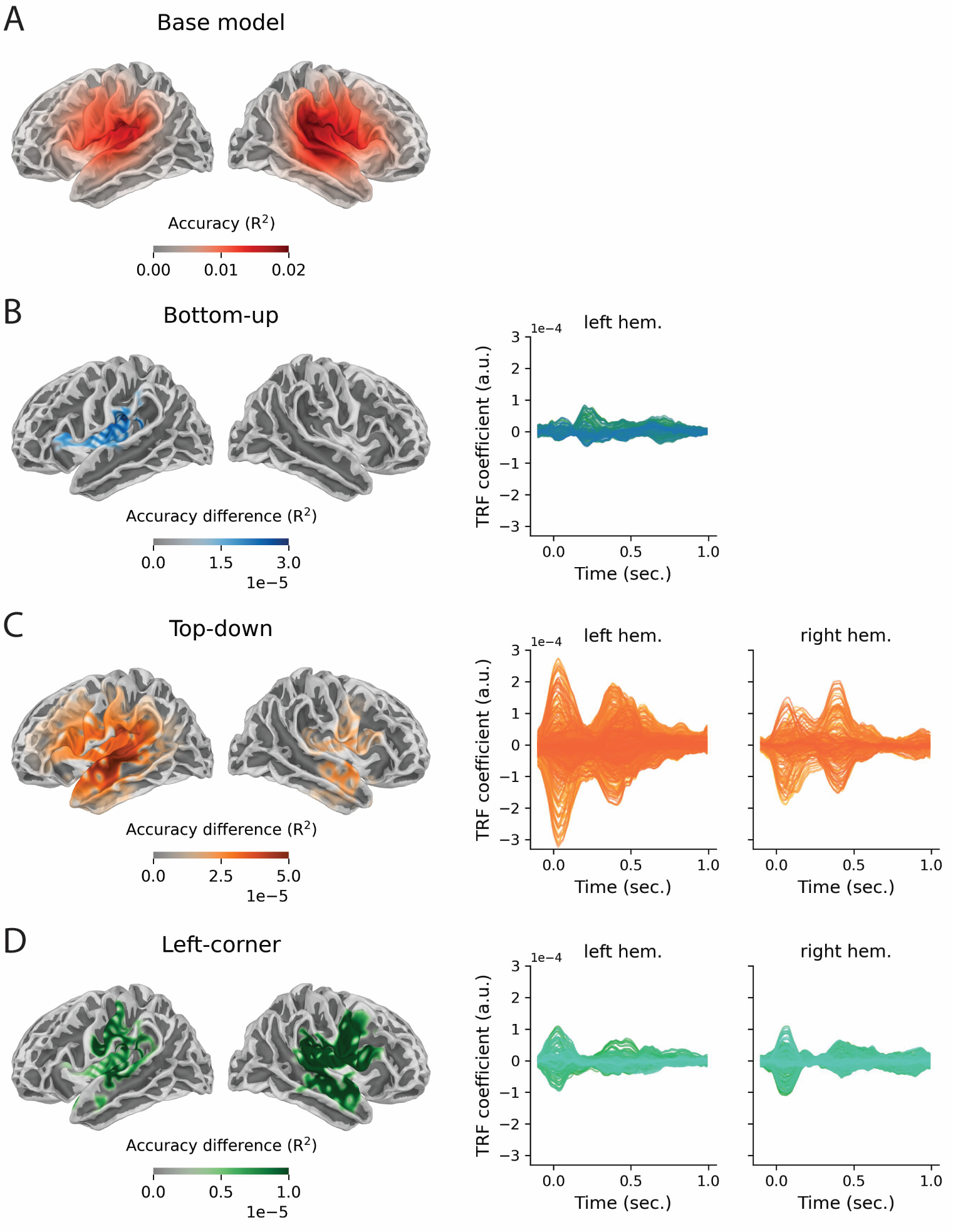

Supplement: S2 Fig — The results for the base model are presented in (A). The results for the syntactic models correspond to the predictors reflecting node count from the bottom-up (B), top-down (C), and left-corner (D) methods. All clusters that were significant at uncorrected alpha = 0.05 are displayed. Notice that the scales of the color bars are different across the plots. The plots on the right show the temporal response functions for node count derived from the bottom-up, top-down, and left-corner parsers in their respective models. Each line reflects the response function in a source point that was part of a cluster which showed a significant improvement in reconstruction accuracy. Data are available on the Radboud Data Repository (https://doi.org/10.34973/m1vp-hc15). (TIFF) [file pbio.3002968.s003.tiff]

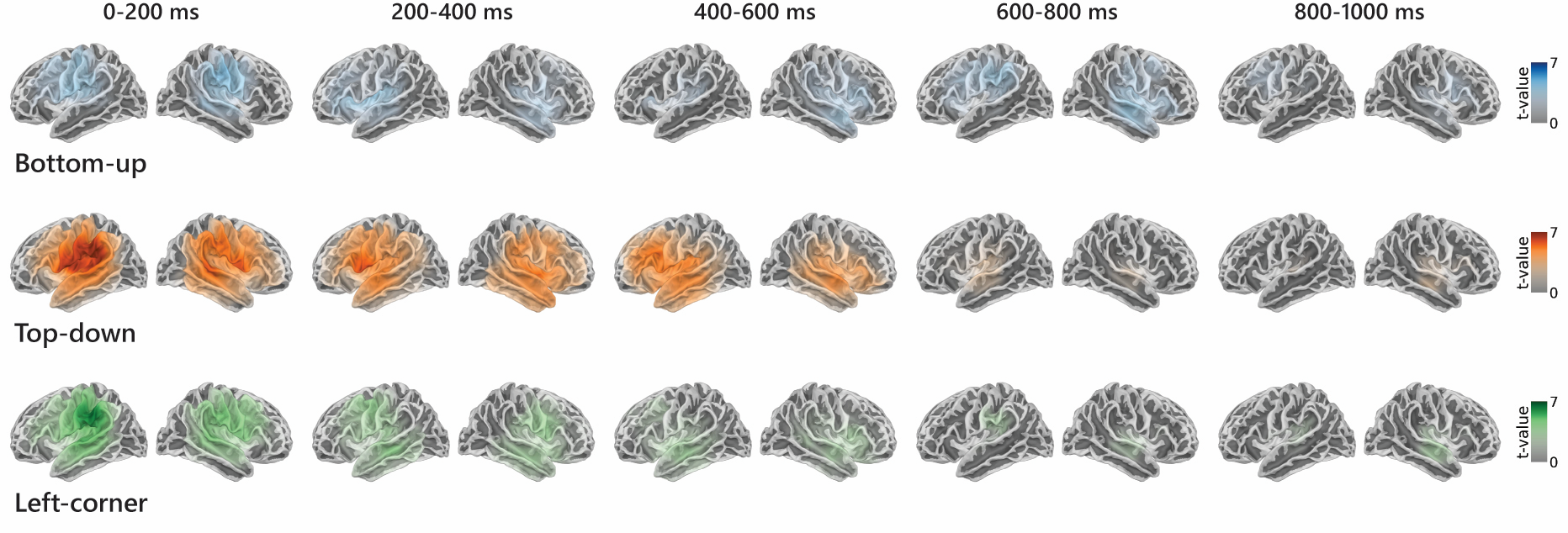

Supplement: S3 Fig — The colors represent t-values in source clusters that were significantly responsive (at corrected alpha = 0.0083) to the predictor in the indicated time windows. The midpoints of the color scales are at 70% of the maximum, such that the colored plots highlight the sources with the strongest effects. Data are available on the Radboud Data Repository (https://doi.org/10.34973/m1vp-hc15). (TIFF) [file pbio.3002968.s004.tiff]

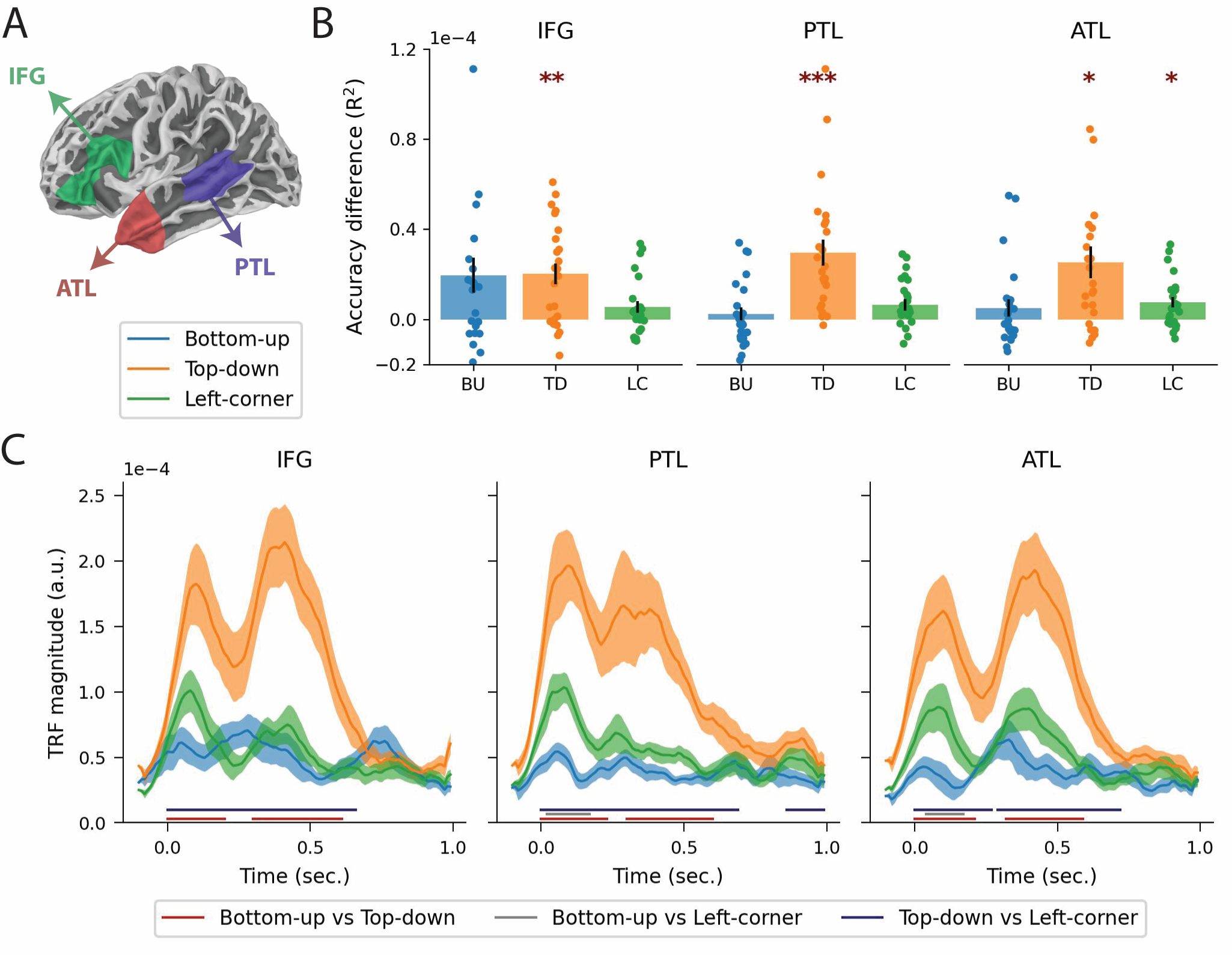

Supplement: S4 Fig — (A) Spatial extensions of the 3 regions of interest. (B) Difference in reconstruction accuracy with the base model, plotted for left IFG, PTL, and ATL. The height of each bar indicates the improvement in reconstruction accuracy when only the relevant syntactic predictor was added to the base model. The drops represent the accuracy difference for individual participants, and the error bars represent the standard error of the mean across subjects. (C) Temporal response functions for node count derived from bottom-up, top-down, and left-corner parsers in their respective models. Error bars reflect the standard error of the mean per time sample. The horizontal bars below the TRFs reflect the temporal extensions of the significant clusters from the pairwise analysis. Data are available on the Radboud Data Repository (https://doi.org/10.34973/m1vp-hc15). (TIFF) [file pbio.3002968.s005.tiff]

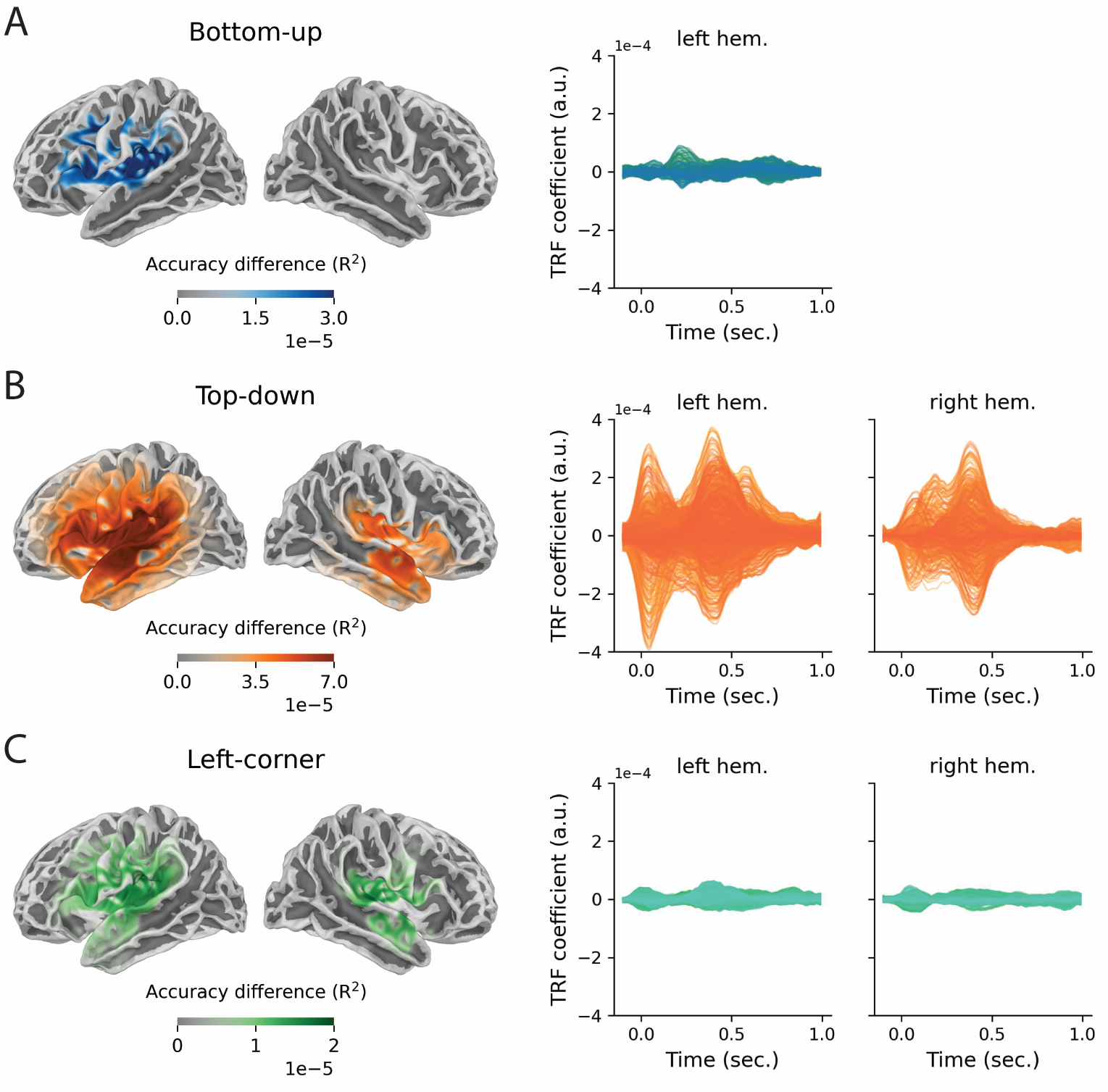

Supplement: S5 Fig — We repeated our analysis with TRF models from which the statistical predictors word frequency, surprisal and entropy were omitted. Results are shown separately for the effects of the bottom-up (A), top-down (B), and left-corner (C) predictors, and reflect the sources of significantly improved explained variance and temporal response functions in significant source points. Significance was determined by comparing the reconstruction accuracy of the full model to the reconstruction accuracy of a null model from which the relevant predictor was omitted. All clusters that were significant at uncorrected alpha = 0.05 are displayed. Notice that the scales of the color bars are different across the source plots. As explained in S1 Text, section 2, this pattern of results is similar to the results in Fig 4 of the main manuscript, showing that the relative effects of the syntactic predictors are stable when word frequency, entropy, and surprisal are omitted. Data are available on the Radboud Data Repository (https://doi.org/10.34973/m1vp-hc15). (TIFF) [file pbio.3002968.s006.tiff]

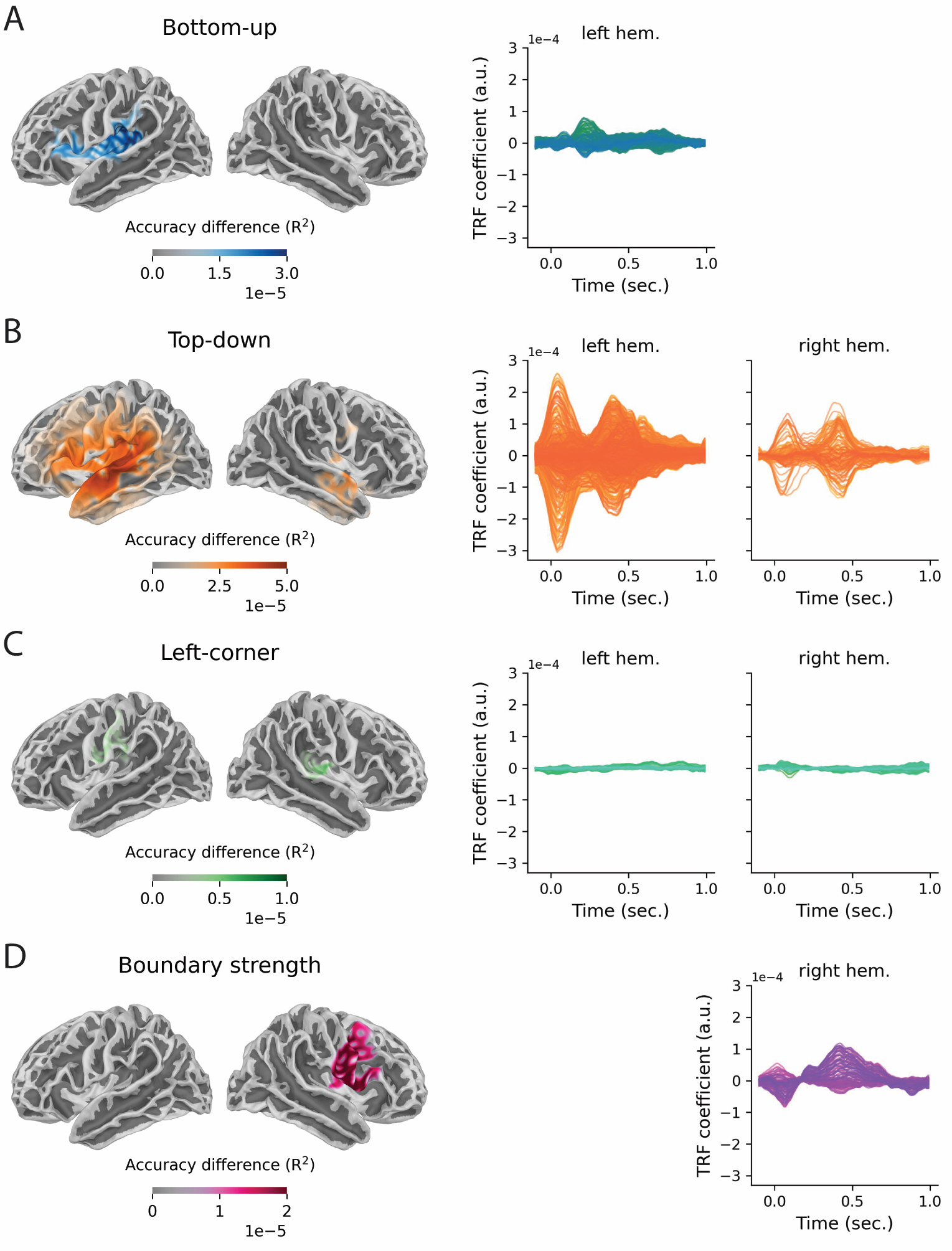

Supplement: S6 Fig — We repeated our analysis with TRF models to which we added a prosodic predictor. The results reflect sources of significantly improved explained variance and temporal response functions in significant source points, shown separately for the effects of the syntactic predictors bottom-up (A), top-down (B), and left-corner (C), and the prosodic predictor boundary strength (D). Significance was determined by comparing the reconstruction accuracy of the full model to the reconstruction accuracy of a null model from which the relevant predictor was omitted. All clusters that were significant at uncorrected alpha = 0.05 are displayed. Notice that the scales of the color bars are different across the source plots. As explained in S1 Text, section 3, this analysis shows that the top-down effect is stable and independent of prosody, and that the variance explained by the bottom-up and left-corner predictors is reduced by the addition of prosodic boundary strength as predictor. Data are available on the Radboud Data Repository (https://doi.org/10.34973/m1vp-hc15). (TIFF) [file pbio.3002968.s007.tiff]

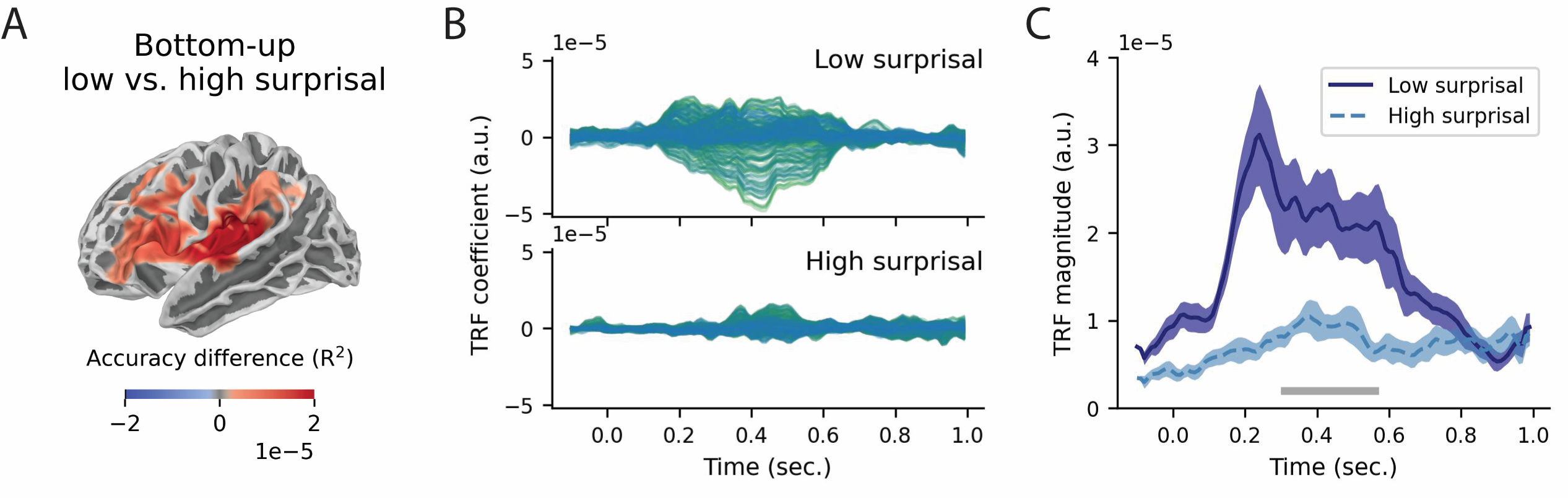

Supplement: S7 Fig — We included an additional analysis in which the effect of integratory structure building was evaluated separately for low- vs. high-surprisal words. (A) Significant sources of the accuracy differences between bottom-up node counts for low- vs. high-surprisal words. The positive accuracy difference indicates that the reconstruction accuracy was higher for the model with low- than for the model with high-surprisal words. (B) Temporal response functions for the bottom-up predictor, estimated separately for high-surprisal and low-surprisal words. Each line reflects the TRF estimated for a source point that was significant in the reconstruction accuracy analysis (i.e., the colored areas in (A)). (C) Amplitude of the response functions of the bottom-up predictor for low- and high-surprisal words, averaged over significant sources. The error bars reflect the standard error of the mean per time sample. The horizontal bar below the TRFs reflects the temporal extension of the largest cluster indicating the significant difference between the TRF amplitudes. Data are available on the Radboud Data Repository (https://doi.org/10.34973/m1vp-hc15). (TIFF) [file pbio.3002968.s008.tiff]
