# Supplementary material for: Language-specific neural dynamics extend syntax into the time domain
Source: PLoS Biol. 2025 Jan 21;23(1):e3002968. doi: 10.1371/journal.pbio.3002968 (PMC11750093; doi:10.1371/journal.pbio.3002968)
Supplement: S1 Table — (PDF) [file pbio.3002968.s009.pdf]

S1 Table. Auditory stimuli.

Story part	Duration
Andersen 1a	4 min 58 sec
Andersen 1b	5 min 17 sec
Andersen 1c	4 min 49 sec
Andersen 1d	5 min 50 sec
Grimm 1a	6 min 6 sec
Grimm 1b	6 min 40 sec
Grimm 2a	5 min 3 sec
Grimm 2b	5 min 32 sec
Grimm 2c	5 min 2 sec
