# Supplementary material for: Language-specific neural dynamics extend syntax into the time domain
Source: PLoS Biol. 2025 Jan 21;23(1):e3002968. doi: 10.1371/journal.pbio.3002968 (PMC11750093; doi:10.1371/journal.pbio.3002968)
Supplement: S2 Table — (PDF) [file pbio.3002968.s010.pdf]

S2 Table. Predictors included in each model.

Model name \ Predictor(s)	Spectrogram/ Onsets	Word onset	Word frequency/ Entropy/ Surprisal	Bottom-up	Top-down	Left-corner
**Base**	X	X	X			
**Bottom-up**	X	X	X	X		
**Top-down**	X	X	X		X	
**Left-corner**	X	X	X			X
