# Supplementary material for: Language-specific neural dynamics extend syntax into the time domain
Source: PLoS Biol. 2025 Jan 21;23(1):e3002968. doi: 10.1371/journal.pbio.3002968 (PMC11750093; doi:10.1371/journal.pbio.3002968)
Supplement: Attachment — Submitted filename: response_to_reviews.pdf [file pbio.3002968.s011.pdf]

Response to reviewers

Reviewer #1:

SUMMARY

The authors report an n = 24 MEG study on the neural correlates of syntactic structure building in the human brain. Using a modeling approach comparing different parsing algorithms, they relate MEG activity in specific time windows in specific brain areas to specific cognitive operations assumed to be performed during syntactic structure building, with the psychological goal of arriving at an interpretable representation of what we call sentences.

The analyses are done very adequately. The authors rightly go all the way from a representational assumption via a parsing algorithm and complexity metric to a state-of-the-art regression model on brain data of high spatio-temporal resolution. The authors' writing shows they know the psycholinguistic reasoning behind the parsing algorithms they are employing, so the whole path from cognitive science to the highest-possible-with-extracranial spatiotemporal resolution is taken. I enjoyed reviewing this manuscript, because this is how I know this type of work must be performed. The standard is very high here and it is an example for the readership who wishes to be inspired.

We thank the reviewer for this positive evaluation!

I list multiple points for further improvement of this impressive work below.

MAJOR

(1) Distribution of age: The age range measured here is rather wide. I think that more efforts need to be taken to estimate the generalizability of the current results and whether they hold beyond young / middle-aged / senior adults. Specific comment:

245: The age range is non-standard. There is evidence that syntactic processing changes quite a bit already around 50, if not earlier. Please comment on this / address by supplementary analysis. I would also like to know how age was distributed across the sample to judge whether the current findings can be taken as representative for adult processing, or whether the findings could even be restricted to the aging brain. In particular with respect to the idea that top-down processes are stronger in the aging brain + the shallow parsing concept / its relationship to age, this could be an interesting additional research question to be addressed, e.g., by correlations with an age predictor. Please comment / consider.

Response: The histogram below shows the age distribution of the participants. The median is 28 years. More than half (13/24) of the participants are below 30, seven participants are older than 35, and four participants are above 50 years old. We now include this information in Section 2.1 of the revised manuscript.

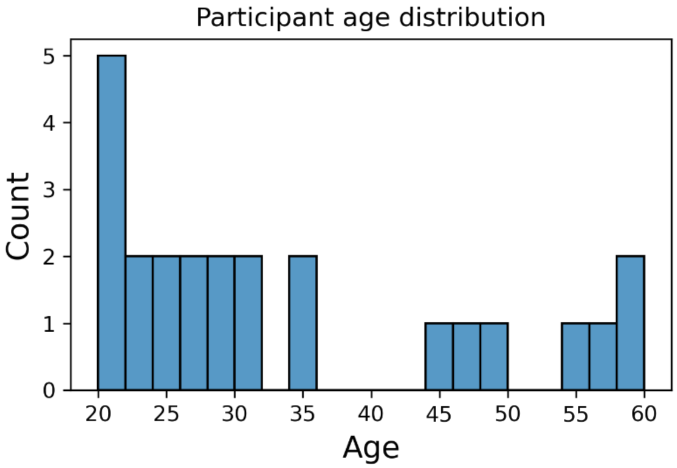

Given that the large majority of the participants is between 20-35 years, which is the standard age range in psycholinguistic and neurolinguistic experiments, we think that our results are representative for (young-)adult sentence processing and can therefore be compared to the existing literature.

To nevertheless check whether the inclusion of the older participants affected the results, we removed all seven participants with age > 35 before running the statistical analysis on the reconstruction accuracies. The reconstruction accuracy plots displayed below (masked for significance) show that the pattern of results for the remaining seventeen younger participants is essentially the same as in the main manuscript, with the top-down predictor explaining most variance in left-hemispheric neural activity.

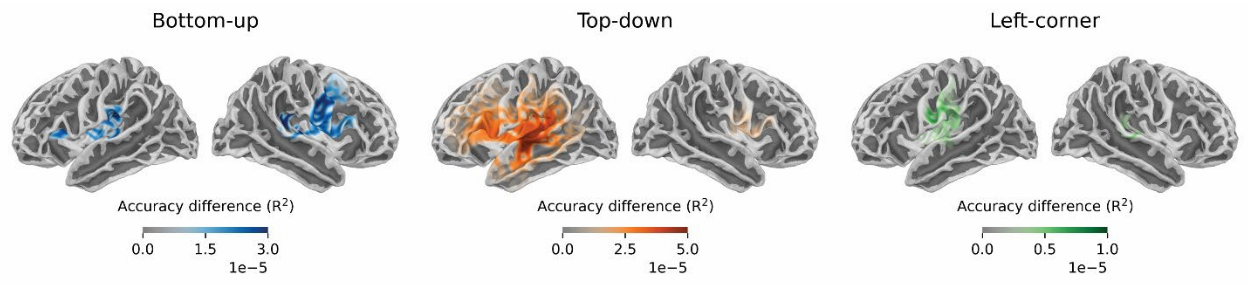

The results below show the same analysis, but then restricted to the twenty participants younger than 50. Again, the pattern of results is the same, meaning that including participants older than is usual did not affect our results.

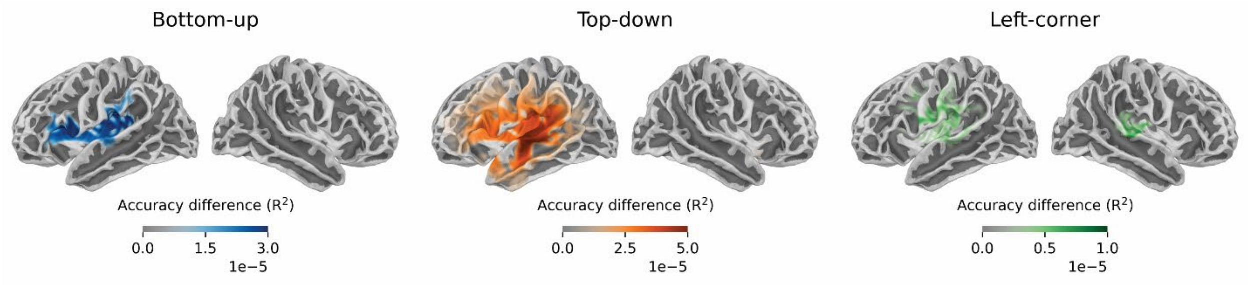

(2) Prosody / boundaries: What is the covariance of node count and prosodic boundary strength? Strong enough to pose a confound? Control analyses should be performed. Specific comment:

351ff: Was there a statistical relationship between the strength of prosodic boundaries and node count? Please provide supplementary analysis on this (e.g., Inbar et al., MEG, 2023,'s recently employed method).

Response: We thank the Reviewer for this comment, which inspired a number of exciting new research questions which we also had converged up on and which we are excited about exploring in future work. In the revised Supplementary Information, we have included additional analyses on the role of prosodic boundaries (Section S4, pages 10-13).

In short, we extracted prosodic boundaries in our audiobook stories using the Wavelet Prosody Toolkit (Suni et al., 2017), which was also used in Inbar et al.'s recent MEG study. This algorithm performs a continuous wavelet transform of a combined signal that is composed of fundamental frequency, energy and word duration, from which a continuous measure of prosodic boundary strength is computed for each individual word. In our stimuli, prosodic boundary strength was positively correlated with bottom-up node count (r = 0.34) and left-corner node count (r = 0.19), but not with top-down node count (r = 0.04). This provides a first indication that our results cannot be explained (entirely) by prosody, because the syntactic feature that explains most variance in neural activity (top-down) has no relationship to prosody. The moderate positive correlation between prosodic boundary strength and bottom-up node count nevertheless warrants additional analyses.

We therefore included prosodic boundary strength as a regressor in our TRF models. This analysis showed that even when prosody was explicitly controlled, the effects of all syntactic predictors remained present (see Figure S4.1). However, the variance explained by bottom-up and left-corner node counts was significantly reduced by the addition of prosodic boundary strength, suggesting that these syntactic features share neural variance with prosody. This relationship between prosodic boundaries and syntactic integration points to a potential role for prosody in supporting the inference of syntactic structure.
We have added the following discussion to Section 4.1 of the revised manuscript:

"Compared to the top-down TRF, bottom-up effects were relatively weak in amplitude. This is quite striking because, of all syntactic features, the bottom-up predictor is most strongly correlated with properties of the acoustic signal. That is, bottom-up node count quantifies syntactic phrase closure, which is related to the closure of prosodic phrases (Ferreira, 1993; Nespor & Vogel, 1986; Selkirk, 1984) and moderately correlated with the strength of prosodic boundaries across all story segments (r = 0.34 in our stimuli). To determine whether any of the

syntactic effects might be explained in terms of brain responses to prosody, which also modulates delta-band activity (Bourguignon et al., 2013; Inbar et al., 2023; Rimmele et al., 2021), we included prosodic boundary strength as predictor in our TRF models (see Supplementary Information S4). The results show that while the top-down effects are quite stable, the unique variance explained by the bottom-up and left-corner predictors is significantly reduced by the addition of prosodic boundary strength as regressor. It thus seems that the integratory effects of bottom-up and left-corner node count are modulated by prosody, pointing to a potential role for prosody in supporting the inference of syntactic structure (Coopmans & Martin, 2023; Degano et al., 2024). The top-down effect, however, is not affected by prosodic boundary strength, providing additional support that it reflects abstract structure building. More generally, this result reinforces the idea, alluded to the introduction, that perception requires the brain to add information to its analysis of the physical environment. Spoken language comprehension is more than passive extraction of information from speech – it is an active inference process, during which the brain adds its knowledge of syntactic structure. Because syntactic effects cannot be driven entirely by what is in the signal, they must at least partially reflect what the brain perceptually infers from that signal.” **(pages 32-33, lines 702-721).**

(3) Refining the search space for time course analysis: Why not mask the time course analysis spatially with the clusters shown in Figure 4 to ditch new areas popping up that are hard to interpret? The authors use the timing of the TRF accuracy for masking, but (if I understand correctly) not the space? Specific comment:

527: The source configurations in this figure differ drastically from those in the previous figure. E.g., "Top-down": How can it be that the top-down parser improves TRF accuracy in the left hemisphere (and a bit right aTL), but then the time courses are all strong in the right hemisphere, too? Why not mask the source analysis of the time courses (Figure 5) with the results / maps shown in Figure 4? This would get rid of areas where the TRF accuracy is not improving.

Response: The Reviewer is correct in noting that the spatial topography of the accuracy analysis is not used to constrain the temporal-spatial analysis of the TRF coefficients. We chose not to constrain the statistical analysis of TRF coefficients because the accuracy analysis is based on the entire TRF window and therefore provides a more conservative measure than the analysis of response functions does. It is quite possible that the TRF coefficients for a given brain region are significantly non-zero at certain lags, even if the increase in reconstruction accuracy for that same brain region is not significant. We therefore think that combining the two analyses, both in a relatively unrestricted way, leverages their strengths and provides the most informative picture.

That being said, we acknowledge that the TRF source results in Figure 5 are remarkably different from the reconstruction accuracy results in Figure 4A. This is likely due to the fact that the t-statistics in Figure 5 were estimated on unrectified TRFs. This leads to cancellation of TRF coefficients on opposite sides of sulci (whose dipoles have opposite orientation) and therefore yields discontinuous clusters, which in turn might give the unwanted impression that there are many distinct neural sources. This problem was brought to our attention by Reviewer 2 and has been fixed in the revised manuscript. As we discuss in our response to that reviewer (their comment #2), we now report t-values derived from a statistical analysis of rectified TRFs. Figure 5 in the revised manuscript shows the resulting t-maps, which are

baseline-corrected by subtracting the average activity at negative lags (i.e., 100 ms preceding t = 0). In contrast to the original Figure 5, which was difficult to interpret, the revised TRF results are much more closely linked to the reconstruction accuracy results. Moreover, they reveal interesting information about the time courses of the different effects. That is, Figure 5 shows that while the top-down TRF is strongest at early lags, the bottom-up TRF has a more temporally spread time course, and the left-corner effect combines these two effects (an early peak in addition to a prolonged time course). We find this result interesting because it aligns with the idea that the mildly predictive left-corner parsing strategy combines properties of top-down and bottom-up parsing.
We have added the following discussion of these results to Section 3.2 of the revised manuscript:

"While these results are informative about the overall strength and timing of the responses, they do not show which regions are involved at which time points. This information is provided in Figure 5, which shows the source t-values (based on one-tailed, one-sample t-tests) of the rectified TRFs of the three syntactic predictors. Non-parametric cluster-based permutation tests were used to determine when and where the TRF coefficients of each syntactic predictor deviated from zero. Because this involves 6 comparisons (3 TRFs * 2 hemispheres), clusters were evaluated at alpha = 0.0083. The results are split up into five time windows (corresponding to different delays in TRF estimation) for the purpose of visualization only. They show that the effect of the top-down predictor is strongest and peaks early (as in Figure 4B), but also that it is distributed rather bilaterally at those early lags. That suggests that predictive structure building, as quantified by top-down node counts, poses strong demands on brain computation, in particular in early time windows. The fact that right-hemisphere activity contributed less to improving reconstruction accuracy might however indicate that these effects are less stable across participants. Another potentially informative observation in Figure 5 is that the left-corner TRF seems to combine aspects of both the top-down and the bottom-up effects. It peaks early, like the top-down TRF, but its time course is prolonged, which is the case for the bottom-up TRF as well. This observation is interesting because it aligns with the idea that the mildly predictive left-corner parsing strategy combines properties of top-down and bottom-up parsing." **(page 26, lines 557-573)**

We also updated the corresponding figure (Figure 2.3) and section in the revised Supplementary Information (Section S2.2; page 5, lines 63-67).

(4) Relating the findings to the structural literature: We do need to link the timing of syntactic processing to the underlying structural neuroanatomy, and the current findings allow for doing so (at least a bit, in the form a discussion).

575ff: The authors should make an effort to relate their results to the structural neuroanatomy of syntax (dorsal & ventral networks, prior proposals on what each network does & how these proposals relate to the cognitive operations modeled in the current submission). Linking the MEG time course world to the structural architecture of syntax is desperately needed in the literature, and the current time courses & source configurations (in particular when making use of my point about masking the Figure-5 analyses with the Figure-4 results, spatially) would allow for some rather solid discussion (I encourage the authors!)

Response: The Reviewer is completely on point about the importance of linking the results in different subfields of the cognitive neuroscience of language. However, as we are sure the Reviewer knows, MEG source estimates unfortunately do not yet offer the resolution necessary to make meaningful links to anatomical structures. Moreover, the anatomical structures involved in syntactic processing are not well enough understood, and progress on this topic requires more extensive structural MRI and DTI data, which we currently do not have. We think that more work from a neuropsychological and intracranial perspective may be necessary before functional neuroimaging times series can be interpreted in the context of neuroanatomical constraints. We do agree with the Reviewer about the importance of this kind of multi-methodological work and therefore added this point to the discussion on page 32 (lines 699-701).

(5) How do the current results relate to the classical ERP/F literature (e.g., ELAN / sMMN)? Can the authors discuss the prediction / integration debate? How do the current results relate to source localizations of these ERPs/Fs and / or the MRI literature using similar paradigms? It is fine to discuss the Matchin review, but it would increase your readership of you would also speak to the psycholinguistics concepts / issues a bit more (in the discussion).

Response: In Section 4.1 of the revised manuscript, we now discuss the effects of top-down node count in the context of the ERP literature. In short, we discuss the fact that the top-down TRF is distinctly bimodal, with an early peak at around 100 ms and a subsequent peak at around 400 ms. Such early effects of syntax have been reported in ERP studies as well, in particular in response to phrase-structure violations (both ELAN and sMMN). In line with the predictive nature of top-down node counts, these early effects have been interpreted in terms of predicted (or 'precompiled') phrase-structure representations. The subsequent peak in the top-down TRF is similar to effects of syntactic surprisal reported in naturalistic paradigms. We interpret it accordingly as a response related to the disconfirmation of predicted structures based on incoming information, which triggers a revision of the initial parse of the sentence. The top-down effect thus has both a predictive component as a well as a somewhat integratory component. This is possible because, even if node counts for a given word are derived from a fully predictive parser, they are assigned to the word itself. This means that a disconfirmation of the predicted structure elicits a response in the time window of that same word.
We have added the following discussion to Section 4.1 of the revised manuscript:

"The first peak around 100 ms was strongest in posterior regions and likely reflects predictive structure building, which can occur in the PTL within the first 150 ms after word onset (Matchin et al., 2017). Indeed, the early timing of this effect is consistent with the predictive nature and temporal spell-out of top-down parsing, which is maximally eager and postulates syntax early in time. Previous electrophysiological studies have shown that phrase-structure violations elicit similarly early responses, including an early left anterior negativity (Herrmann et al., 2011a, 2011b; Jakuszeit et al., 2013) and a syntactic Mismatch Negativity (Hasting et al., 2007; Herrmann et al., 2009), which have been linked to left superior temporal cortex in lesion and localization studies (Herrmann et al., 2009, 2011a, 2011b; Jakuszeit et al., 2013). The fact that phrase-structure violations are detected so early strongly suggests that the brain generates predictions about the syntactic properties of upcoming material, perhaps in the form of precompiled phrase structure representations (Hagoort, 2005; Hasting et al., 2007; Herrmann et al., 2011a). The second, later peak in the top-down TRF was strongest in more anterior

regions and is largely consistent with syntactic surprisal effects, both in terms of timing (Brennan & Hale, 2019; Hale et al., 2018; Heilbron et al., 2022) and spatial distribution (Henderson et al., 2014). It might therefore reflect responses related to the disconfirmation of predicted structures based on incoming information, which triggers a revision of the initial parse of the sentence." **(page 32, lines 678-694)**

MINOR

1-20: "yet, syntactic rules are" pls. change to *are widely assumed to be* or *many theories of human language* or something else, as this is not an established fact. No one really knows whether and how and where syntactic representations exist in the physical & cognitive sense, in particular whether there are atemporal representations (e.g., a whole tree of a whole sentence being present in the mind/brain in a given instant).

Response: We have changed this in line with the Reviewer's suggestion.

28: "words hierarchically are" pls. change this to be non-factual. The authors should write that *leading theories in theoretical linguistics assume that words are...* and so on. Again, these are not facts, but hypotheses / theories.

Response: We have changed this accordingly, thank you.

29f: "are abstract and not bound" see previous comments. These are hypotheses / theories.

Response: We have added "these theories assume that…".

32: Introduce term "parsing" to the non-experts, e.g., "The incremental buildup of syntactic structure over time is termed parsing in psycholinguistics".

Response: We have added a sentence introducing the term "parsing".

33: "dynamics" -> "temporal dynamics" or "word-by-word dynamics"

Response: We have added the word "temporal".

36: I am not sure that "linguistic theory" is at the "computational level". The authors said that they assume that the initial input (production) and final output (comprehension) are an atemporal representation of syntactic structure. Now how is this computational? In my understanding, the buildup of this structure over time is computational, no?

Response: We understand the confusion and thank you for bringing it to our attention. The word "computational" in this sentence refers to Marr's (1982) tripartite architecture, in which the computational level is concerned with the nature of the capacity being described. As such, it does not refer to input- our output processes, which are described at the algorithmic level. Here, we follow the common idea that linguistic theories are formulated at the computational level, while psycholinguistic theories of language processing are algorithmic-level theories (e.g., Embick & Poeppel, 2015; Marantz, 2005; Marr, 1982; Sprouse & Lau, 2013). In a computational-level theory of syntax, the word 'computational' can be used to refer to

derivations of syntactic structures (which are often logically ordered). But because the entire derivational analysis is atemporal, the term 'computational' does not refer to the actual processes that build up this structure over time. The latter belongs to the algorithmic level. To make clear that this discussion is couched in terms of Marr's distinction between the computational, algorithmic and implemental levels of analysis, we added a reference to Marr (1982) to the relevant section.

Embick, D., & Poeppel, D. (2015). Towards a computational(ist) neurobiology of language: Correlational, integrated and explanatory neurolinguistics. *Language, Cognition and Neuroscience*, *30*(4), 357–366. https://doi.org/10.1080/23273798.2014.980750

Marantz, A. (2005). Generative linguistics within the cognitive neuroscience of language. *The Linguistic Review*, *22*(2–4), 429–445. https://doi.org/10.1515/tlir.2005.22.2-4.429

Marr, D. (1982). *Vision: A computational investigation into the human representation and processing of visual information*. W. H. Freeman & Co.

Sprouse, J., & Lau, E. F. (2013). Syntax and the brain. In M. Den Dikken (Ed.), *The Cambridge Handbook of Generative Syntax* (pp. 971–1005). Cambridge University Press. https://doi.org/10.1017/CBO9780511804571.033

43: Bever & Poeppel (2010) should be added to the references here I believe.

Response: We have added this reference.

54: "has to be" -> "is assumed to be built when presupposing that the input / output [...] a hierarchical structure built from [...]"

Response: We have changed this into "…structure that is assumed to be built…".

74: I am not sure whether a complexity metric quantifies a "states". To me, a state of sth. qualitative (as in: one state compared to another state). Can the authors say rather that the metric quantifies the magnitude of a state / how strong a given state is assumed to be by the hypothesis?

Response: We do not think that states are necessarily qualitative. Here, we follow Brennan's (2016) introduction of neuro-computational language models, in which he writes: "a sequence of mental states that connect the linguistic input and desired output. A complexity metric *C* quantifies this sequence of mental states in some way" (p. 302). In line with the Reviewer's suggestion, we have changed the corresponding sentence into: "The complexity metric *C* represents the magnitude of the neural state".

76: What is meant by "relevance"? Please unpack.

Response: The term "relevance" here is synonymous with "reality" or "realization", in the sense that it is possible to determine the psychological or neural reality of theoretical-linguistic constructs by evaluating to what extent they explain variance in brain activity (i.e., how are these constructs realized in neural activity?). To make this clear, we have changed "cognitive and neural relevance" into "cognitive and neural realization".

79: What is "linguistic competence"? Do the authors mean "acquired knowledge of syntactic structure / rules"?

Response: Yes. To avoid the term "competence", we changed it into "knowledge of linguistic structure" (we use "linguistic structure" instead of "syntactic structure" because the general method applies to all types of linguistic structures).

86f: This is unclear to me. What are "natural language structure" and, in particular, where are these (mind/brain/textbook)? I understand the "performance" bit better (~ words are incrementally integrated into some kind of syntactic structure on the fly), but what does this have to do with some kind of "competence" / how would this "competence" be different, physiologically / cognitively? If the authors think this is important, they should (a) unpack their reasoning a little more and (b) explain to the non-experts a bit about the historical genesis of the dualism of competence / performance and why it is relevant, in their view, to understanding the neural implementation of syntactic processing.

Response: We understand the confusion of this statement, so we should give some background. Linguistics has often been criticized for not being mentalistic – that is, not creating theories about (linguistic) cognition. In the past decade, this view has been reinvigorated by several authors (e.g., Frank & Bod, 2011; Frank et al., 2012), who argued that representations of syntactic structure (classically referred to as models of 'competence') are merely "theoretical constructs", whose properties are not necessary to explain how people process language (classically referred to as 'performance'). Against this background, we added the corresponding sentence to show that we disagree with this view, as we see a strong relation between competence (i.e., knowledge of language and linguistic structures) and performance (i.e., our use of language in concrete situations). To avoid unintended interpretations of that sentence though, we have slightly rephrased it and removed the words competence and performance.

Frank, S. L., & Bod, R. (2011). Insensitivity of the human sentence-processing system to hierarchical structure. Psychological Science, 22(6), 829–834. https://doi.org/10.1177/0956797611409589

Frank, S. L., Bod, R., & Christiansen, M. H. (2012). How hierarchical is language use? Proceedings of the Royal Society B: Biological Sciences, 279(1747), 4522–4531. https://doi.org/10.1098/rspb.2012.1741

94: Motivate use of one particular linguistic theory, pls. Why is this theory a better theory than others / why did the authors choose it instead of other options?

Response: We chose to construct X-bar tree structures because X-bar theory is appropriately expressive to deal with natural language structures. That is, they can naturally capture the different types of hierarchical dependencies found in natural languages, like unbounded dependencies and displacement dependencies. That being said, by using X-bar theory we do not intend to argue that this is the only 'correct', accurate, or sufficient grammatical formalism, nor should our results be taken as neurobiological evidence for X-bar theory. As the Reviewer likely well knows such that they are motivated to ask this question, there are other types of grammars that also have the right level of expressivity to model natural language syntax (i.e., slightly beyond context-free power), and there is important recent work that adopts these grammars in neuro-computational language models. For instance, fMRI studies by Stanojević et al. (2021, 2023) showed that node counts derived from structures

generated by Combinatory Categorial Grammars improve predictive accuracy in regions of the left-hemispheric language network above and beyond predictors derived from large language models and context-free phrase structure.

We have added this motivation for our use of X-bar theory to Section 1.1.1 of our revised manuscript, where we cite relevant studies that have used different types of grammars (X-bar theory, Minimalist Grammars, Combinatory Categorial Grammars) in neuro-computational language models:

"We chose to construct X-bar tree structures because they are appropriately expressive to deal with natural language structures, which is to say that they can capture the different types of hierarchical dependencies found in natural languages (e.g., long-distance dependencies, displacement, see Section 2.3). Indeed, recent work using neuro-computational models has shown that complexity metrics derived from these and similar kinds of structures predict brain activity above and beyond activity accounted for by both sequential models and models that are hierarchical but less expressive (Brennan et al., 2016; Li & Hale, 2019; Nelson et al., 2017; Stanojević et al., 2023)." **(page 6, lines 95-102)**

Stanojević, M., Bhattasali, S., Dunagan, D., Campanelli, L., Steedman, M., Brennan, J., & Hale, J. (2021). Modeling incremental language comprehension in the brain with Combinatory Categorial Grammar. *Proceedings of the Workshop on Cognitive Modeling and Computational Linguistics*, 23–38. https://doi.org/10.18653/v1/2021.cmcl-1.3

Stanojević, M., Brennan, J. R., Dunagan, D., Steedman, M., & Hale, J. T. (2023). Modeling Structure-Building in the Brain With CCG Parsing and Large Language Models. *Cognitive Science*, *47*(7), e13312. https://doi.org/10.1111/cogs.13312

96: "natural language structures" Please explain to the non-expert reader why "movement" is such a structure and possibly also what movement is.

Response: In our revised manuscript, we explain movement in Section 2.3 (page 15, lines 302-314), where we introduce traces. In the sentence to which the Reviewer's comment refers, we added "see Section 2.3" to refer to this additional explanation. We also replaced "movement" by "displacement", which is a theory-neutral term to describe the phenomenon (of which "movement" is a particular theoretical analysis).

166: Unpack for non-experts please—what is a complement?

Response: We have replaced the word "complement" by the less technical term "object", which is also accurate in this context and more explanatory for non-linguists.

237: Somewhat fuzzy. Effects in the literature start at ~80 ms (Herrmann et al.'s MEG, ca. 2009) and continue all the way until the P6 / CPS time windows (i.e., beyond 500 ms, considering reanalysis / model update / syntactic prediction error to be part of "structure building")... Maybe reformulate a bit to mark that your hypotheses are for the immediate word-by-word integrative / predictive parts (*first stage*)?

Response: That is correct. This hypothesis was based on syntactic effects in the few studies that used a naturalistic paradigm. In line with the Reviewer's suggestion, we now emphasize

that our expectations are mainly about the immediate stages of predictive and/or integratory structure building, but that we also consider the possibility that later effects of syntactic reanalysis show up.

252f: Syntax in these stories, some > 150 years old, is certainly different from current syntax of Dutch. The authors should comment on this / address this. That is, not all structure in these stories can be taken to be "naturally occurring".

Response: It is correct that the stories are rather old, but the sentence structures are natural relative to the syntax of modern Dutch. This is because Dutch syntax has undergone only minimal changes in the past 150 years, which can be seen (and analyzed quantitatively) in the recently published historical corpus of written Dutch (Piersoul et al., 2021). The sentence structures in the stories can therefore be considered naturally occurring and are easy to understand.

Piersoul, J., Troij, R. D., & Velde, F. V. de. (2021). 150 years of written Dutch: The construction of the Dutch Corpus of Contemporary and Late Modern Periodicals. *Nederlandse Taalkunde*, *26*(3), 339–362. https://doi.org/10.5117/NEDTAA2021.3.002.PIER

377f: It is a bit unclear to me how word-level complexity metrics are derived from GPT. Depending on the tokenizer, the probabilities delivered by GPT are not for each word, but for each sub-word token. Please comment.

Response: The Reviewer is correct. GPT-2 calculates surprisal for sub-word tokens, which in the majority of cases correspond to single words. However, long words are tokenized into multiple sub-word tokens. In those cases (490 words, 5.73% of the entire stimulus set), we used the estimated surprisal value of the first token as an index of the surprisal of the entire word. This tokenization step does not affect the estimation of entropy, because the entropy at a given word is not specific to that word (or token); it is the uncertainty before observing any words based on the preceding words (or tokens). We have added this explanation to Section 2.7 (page 20, lines 424-428).

408: I am not sure why such a long time window is chosen. (1) The window includes a substantial number of next-words, (2) the complexity metrics are already contextual, so the TRF calculations and the metrics may interact in some way the longer the deconvolution time window is? Please consider / comment.

Response: We chose such a long time-window because we are interested in syntactic structure building, which can elicit responses up to 1000 ms after critical-word onset (as in the classical P600 studies; Hagoort et al., 1993). In line with the ERP literature, we find that the syntax-related responses are not restricted to early time points but also show up at later lags. This is also observed in other studies from our lab that use TRF analysis to model the time course of syntactic processing in naturalistic language comprehension (Slaats et al., 2024; Weissbart & Martin, 2024). For instance, Weissbart and Martin (2024) found that integratory syntactic features have a temporally spread impact on the neural signal, with the largest TRF coefficients at late time lags (> 700 ms).

It is not a problem that this time window includes a number of next words, because the TRF analysis allows controlling for overlapping responses to events that are close in time. During deconvolution, the model tries to estimate only the neural response time-locked to the stimulus presented at time *t* (e.g., Brodbeck et al., 2023). Responses elicited by words at *t* + 1, t + 2, etc. will not affect the estimation of the TRF coefficients if those responses are not systematically related to the feature presented at *t*. This can be seen, for instance, in the TRFs of acoustic onsets, which are known to elicit a transient neural response in bilateral auditory cortex within the first few hundred milliseconds (e.g., Brodbeck et al., 2018, 2020). The figure below shows the TRFs of acoustic onsets, extracted from the full model and averaged across all sources in the left and right hemisphere separately. It shows that, after the initial peak, the response flattens, meaning that acoustic onsets do not explain variance in brain activity at those later time points. This result is not affected by the fact that we chose a long time-window that includes the acoustic onsets of subsequent words.

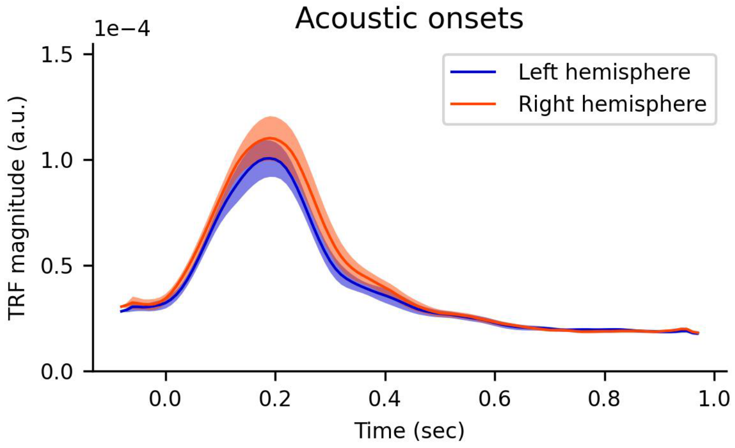

Brodbeck, C., Jiao, A., Hong, L. E., & Simon, J. Z. (2020). Neural speech restoration at the cocktail party: Auditory cortex recovers masked speech of both attended and ignored speakers. *PLOS Biology*, *18*(10), e3000883. https://doi.org/10.1371/journal.pbio.3000883

Brodbeck, C., Hong, L. E., & Simon, J. Z. (2018). Rapid transformation from auditory to linguistic representations of continuous speech. *Current Biology*, *28*(24), 3976-3983.e5. https://doi.org/10.1016/j.cub.2018.10.042

Brodbeck, C., Das, P., Gillis, M., Kulasingham, J. P., Bhattasali, S., Gaston, P., Resnik, P., & Simon, J. Z. (2023). Eelbrain, a Python toolkit for time-continuous analysis with temporal response functions. *eLife*, *12*, e85012. https://doi.org/10.7554/eLife.85012

Hagoort, P., Brown, C., & Groothusen, J. (1993). The syntactic positive shift (sps) as an erp measure of syntactic processing. *Language and Cognitive Processes*, *8*(4), 439–483. https://doi.org/10.1080/01690969308407585

Slaats, S., Meyer, A., & Martin, A. E. (2024). Lexical surprisal shapes the time course of syntactic structure building. Neurobiology of Language. Advance publication. https://doi.org/10.1162/nol_a_00155

Weissbart, H., & Martin, A. E. (2024). *The Structure and Statistics of Language Jointly Shape Cross-frequency Neural Dynamics During Spoken Language Comprehension* (p. 2023.10.06.561087). bioRxiv. https://doi.org/10.1101/2023.10.06.561087

704: "ability [...] dependent on the [...] grammatical properties" Please unpack. It is rather that (a) languages have certain grammatical properties, (b) these properties pose certain cognitive tasks, (c) different languages have different properties, hence magnitudes of different cognitive tasks are different across languages, (d) people are trained on specific languages / grammatical properties, (e) people are skilled to process particular languages, (f) brain responses depend on experience of people with languages (e.g., people have learnt that in language X, making predictions helps processing, whereas in language Y, making predictions is not helpful.).

Response: The relevant sentence in the original manuscript was meant to capture roughly all of these possibilities, which are strongly related and not mutually exclusive. The idea we want to convey is that different languages can pose different demands on certain cognitive processes, because these languages have different (grammatical) properties. Listeners who have experience with a language will therefore rely more on those processes that are suitable for that language. To give an example, while experiments on head-initial structures commonly find that processing costs are increased when the distance between the head and its dependents is increased (Gibson, 1998; Bartek et al., 2011), studies that investigate head-final structures have reported the exact opposite. They find a facilitation in processing of words integrating longer dependencies, an effect that is typically attributed to an increase in the prediction for the upcoming head (Konieczny, 2000; Husain et al., 2014; Nakatani & Gibson, 2008). Language-dependent findings such as these might be linked to the different properties of head-initial and head-final languages. Because the average dependency lengths in head-final languages are considerably longer than those in head-initial languages (Futrell et al. 2015), speakers of head-final languages may be more practiced and therefore more skilled at processing longer dependencies.
We have added a summarized version of these points to Section 4.1 of the revised manuscript:

"Language-dependent findings such as these reinforce the idea that people's ability to generate syntactic predictions might be dependent on the specific grammatical properties of the language. Because the average dependency lengths in head-final languages are longer than those in head-initial languages (Futrell et al., 2015), speakers of head-final languages have more experience with processing longer dependencies and might therefore rely more strongly on a processing strategy that facilitates the comprehension of these structures."
(pages 35-36, lines 783-789).

Bartek, B., Lewis, R. L., Vasishth, S., & Smith, M. R. (2011). In search of on-line locality effects in sentence comprehension. *Journal of Experimental Psychology: Learning, Memory, and Cognition*, *37*(5), 1178. https://doi.org/10.1037/a0024194
Futrell, R., Mahowald, K., & Gibson, E. (2015). Large-scale evidence of dependency length minimization in 37 languages. *Proceedings of the National Academy of Sciences*, *112*(33), 10336–10341. https://doi.org/10.1073/pnas.1502134112
Gibson, E. (1998). Linguistic complexity: Locality of syntactic dependencies. *Cognition*, *68*(1), 1–76. https://doi.org/10.1016/S0010-0277(98)00034-1

Husain, S., Vasishth, S., & Srinivasan, N. (2014). Strong expectations cancel locality effects: Evidence from Hindi. *PLOS ONE*, *9*(7), e100986. https://doi.org/10.1371/journal.pone.0100986
Konieczny, L. (2000). Locality and parsing complexity. *Journal of Psycholinguistic Research*, *29*(6), 627–645. https://doi.org/10.1023/A:1026528912821
Nakatani, K., & Gibson, E. (2008). Distinguishing theories of syntactic expectation cost in sentence comprehension: Evidence from Japanese. *Linguistics*, *46*(1), 63–87. https://doi.org/10.1515/LING.2008.003

730: For the non-experts, the authors might want to explain what traces are; see my above point about movement.

Response: In our revised manuscript, we now explain in Section 2.3 (page 15, lines 302-314) what traces of movement are. In the sentence to which the Reviewer's comment refers, we added "see Section 2.3" to refer to this additional explanation.

Reviewer #2:

The manuscript "Neural dynamics express syntax in the time domain during natural story listening" describes an experiment in which participants were listening to unaltered, naturally read stories in Dutch while neural activity was recorded using MEG. The authors use regression analyses to isolate neural signal variance that can be attributed to syntactic processing. Since syntax is abstract and atemporal, they use a proxy measure, incremental node count, to translate from different models of syntactic structure building to neural responses in the time domain. The different models build the same syntactic structure but differ in when syntactic nodes are added to the tree, either predictively (top-down), adding nodes to the tree before all child nodes are received, or bottom-up, once all the child nodes are received (+ an intermediary 'left-corner'-strategy). Although all models build the same syntactic tree with the same number of nodes, the time at which nodes are added to the tree differs, thus the measure 'incremental node count' is high/low at different parts within a sentence.

This strategy (using a processing difficulty or load measure for studying syntactic structure building during natural speech) has been used in previous studies on this topic, as the manuscript acknowledges. The novelty of the current study, according to the authors, lies firstly in the more comprehensive inclusion of control predictors (acoustic and word-level predictors) and secondly in the finding that top-down syntactic structure building explains a larger part of the neural variance compared to the other models: a finding that is in contrast to similar studies with English material. The authors attribute this to a difference in word-order between English and Dutch which render predictive structure building a more efficient strategy in Dutch.

As a reader whose research focus is not on syntax, I really appreciated the very clear introduction and layout of the research problem. Studying sentence processing using explicit and transparent models, as the current paper does, promises to be very insightful and deserves to be published to the attention of a broad audience, especially as an alternative to the currently prominent approaches using black-box algorithms like large language models. However, I see some problems

with the current paper which would need to be addressed to see whether the main claims of the paper are supported.

1) Effect of control predictors on the model comparison

Since this study is using unaltered natural speech material, it has to deal with the problem that different linguistic predictors are correlated. The authors' strategy is to add control predictors to their regression model. This means that e.g. in Figure 4, the additional R2 for the bottom-up model represents the unique variance explained by the bottom-up model, over and above what can be explained by the combination of acoustic predictors, word-level predictors (frequency & GPT2 surprisal) and the other two syntactic models. This is done to strengthen the evidence that explicit syntactic models explain neural activity above and beyond competing models such as GPT2:

Page 28, line 597: "the null models also contained information-theoretic predictors (e.g., surprisal, entropy) that partially reflect syntactic information and therefore explain some of the variance in brain activity whose origin is syntactic. By including these semi-syntactic control predictors, we stacked the cards against us and likely underestimated the 'true' neural response to syntactic structure"

I agree and it strengthens the basic finding that the neural activity described reflects syntactic structure building. However, the control predictors might bias the comparison between the three syntactic models due to correlations with the syntactic predictors. Looking at the correlation table in Figure S2.1, it seems that the top-down regressor is less correlated with the control regressors than bottom-up or left-corner regressor. So it could be that GPT2 surprisal is correlated more to bottom-up node count and thus decrease the neural variance explained by that model. The correlations with GPT2 entropy/surprisal are overall small, so this might be not be a big problem. But given that the unique neural variance explained is small (e.g. unique R2=5e-05 for the top-down model), it is possible that there is a bias. Does the top-down model still outperform the other models when GPT2-based regressors are removed? A change in effect would be quite problematic for the claims of the paper, especially because GPT2 is a black box model, so it would be quite difficult to explain theoretically.

The correlations are even stronger with the word frequency regressor: .33 for bottom-up, -.081 for top-down. Where does this come from? Looking at the example sentence in Figure 2: Under the top-down model, high node counts are assigned to the beginning of phrases which in this case are mostly function words (with high frequency) and under the bottom-up model, high node counts fall more on content words (with lower frequency); an example phrase being "een doordringende kreet". Is this a pattern that is representative of the corpus? Does this affect the main findings of the paper: namely is the higher R2 of the top-down model due to the inclusion of the control regressor word frequency? Do the other papers in the field use these control regressors, and can the divergent findings be due to that difference, rather than a difference in language Dutch vs. English? Without addressing these questions it's difficult to make comparisons to other papers, or claims about language-related differences.

Response: We completely agree and thank the Reviewer for pointing out that the bottom-up predictor is more strongly correlated with the statistical predictors (in particular, word frequency) than the top-down predictor is. However, we do not think that this explains our results. First, it is not the case that Dutch phrases rarely start with (lower-frequency) content

words. In the Reviewer's example, for instance, the words *doordringende kreet* "piercing cry" form a noun phrase, starting with a low-frequency adjective. Other common cases are verb-initial verb phrases in main clauses. The relationship between high-frequency (function) words and top-down node count is therefore not as consistent as our example in Figure 2 might suggest, and this is corroborated by the effectively non-existent correlation between word frequency and top-down node count (r = -0.081). By contrast, it is correct that Dutch phrases rarely end in (high-frequency) function words (relatively rare cases include stranded prepositions, phrase-final auxiliary verbs, etc.), which might partially explain the correlation between word frequency and bottom-up node count.

In order to determine if this asymmetry can explain the effects we observe, i.e., whether the bottom-up predictor explains less variance than the top-down predictor because bottom-up node count is more strongly correlated with the statistical predictors, we repeated our analysis using TRF models that included no statistical predictors. In our new analysis, the 'full' model included the acoustic spectrogram, the acoustic onsets spectrogram, word onset, and the three syntactic predictors. We then determined the unique contribution of each syntactic predictor by comparing the reconstruction accuracy of the full model to the reconstruction accuracy of a null model from which only one of the predictors was omitted. If the pattern of results of this analysis is similar to the results reported in Section 3.1 of the main manuscript (i.e., the relative effects are stable when entropy, surprisal, and word frequency are taken out), it indicates that the control predictors do not bias the comparison between the three syntactic models.
As we now show in Section S3 of the revised Supplementary Information (pages 8-10), the top-down model still outperforms the other two models when all statistical predictors are removed. In fact, if we compare these effects to the effects of the syntactic predictors when the statistical predictors are included (the original results in Section 3.1), it becomes clear that the removal of the statistical predictors has the largest effect on the top-down and left-corner predictors; the effect of the bottom-up predictor remains roughly the same. That means that the correlation between bottom-up and word frequency, and the inclusion of word frequency as control regressor, does not explain why bottom-up effects are weaker than the effects of top-down.
We have added the following discussion to Section 4.1 of the revised manuscript:

"To determine to what extent our syntactic effects are influenced by the presence of these control predictors, we ran an additional analysis with TRF models from which surprisal, entropy and word frequency were omitted. Removing these statistical predictors increases the variance explained by each syntactic predictor, but the pattern of results remains unchanged: the top-down model still outperforms the two other parsing models (see Supplementary Information S3). The fact that the effects of syntactic processing are robust and stable, both in the presence and in the absence of statistical control predictors, underscores the relevance of syntax for the neural mechanisms underlying language comprehension (Weissbart & Martin, 2024)." **(pages 30-31, lines 647-654)**

2) Temporal response functions on the source level - polarity

The source maps of the TRFs (Figure 5) are quite difficult to digest, compared to the other results. Is it correct that you are estimating the t-statistics from the unrectified TRFs? The problem with MEG source maps is the spatial spread: e.g. the activity of a ground truth source in the superior temporal

gyrus would spread to insula, the STS and the opposite side of the Sylvain fissure on the frontal/parietal operculum, or even further depending on how strong the activity of the ground truth source is. When an unsigned value is plotted such as the accuracy of a regression model (as in Figure 4) this is less problematic, because the activation then appears as one statistical cluster with a focus on the STG (Figure 4B). When a signed value is plotted such as a TRF, and the source model was computed with surface normal orientation constraint, discontinuities appear in the map: the same ground truth source would appear e.g. as a negative activation on the STG and as a positive activation on the opposite of the Sylvain fissure, because the dipoles modeled on those two sides of a sulcus have opposite orientations.

Do the authors think that all these positive and negative clusters in time represent distinct neural sources? If yes, they should be discussed and interpreted. In the current manuscript they are not. The interpretation of the time course of neural activity in the discussion section (page) comes from the time courses plotted in Figure 4 and 6, namely the early and late peak of the top-down model, and the peak of the bottom-up model in-between. This can be nicely seen in Figure 4. But Figure 5 is difficult to interpret. My suggestion would be to rectify the TRF time-series for plotting on the source maps (which is more common in MEG papers), this would potentially lead to larger more contiguous clusters, which can more easily be interpreted as one or a small number of sources + spatial spread. Or drop this figure if it is not interpreted.

Response: We thank the Reviewer for these valuable suggestions. It is correct that we initially estimated the t-statistics from unrectified TRFs, which indeed leads to cancellation of TRF coefficients on opposite sides of sulci and therefore yields discontinuous clusters, which in turn might give the impression that there are many distinct neural sources. To avoid these pitfalls, and to prevent the unwanted interpretation that the positive and negative clusters reflect functionally different effects, we now report t-values derived from a statistical analysis of rectified TRFs. Figure 5 in the revised manuscript shows the resulting t-maps, which are baseline-corrected by subtracting the average activity at negative lags (i.e., 100 ms preceding t = 0). In contrast to the original Figure 5, which was difficult to interpret, the revised TRF results are much more closely linked to the reconstruction accuracy results. Moreover, they reveal potentially interesting information about the time course of the different effects. That is, Figure 5 shows that while the top-down TRF is strongest at early lags, the bottom-up TRF has a more temporally spread time course, and the left-corner effect combines these two effects (an early peak in addition to a prolonged time course). We find this result interesting because it aligns with the idea that the mildly predictive left-corner parsing strategy combines properties of top-down and bottom-up parsing.
We have added the following discussion of these results to Section 3.2 of the revised manuscript:

“While these results are informative about the overall strength and timing of the responses, they do not show which regions are involved at which time points. This information is provided in Figure 5, which shows the source t-values (based on one-tailed, one-sample t-tests) of the rectified TRFs of the three syntactic predictors. Non-parametric cluster-based permutation tests were used to determine when and where the TRF coefficients of each syntactic predictor deviated from zero. Because this involves 6 comparisons (3 TRFs * 2 hemispheres), clusters were evaluated at alpha = 0.0083. The results are split up into five time windows (corresponding to different delays in TRF estimation) for the purpose of visualization only. They show that the effect of the top-down predictor is strongest and peaks early (as in Figure 4B),

but also that it is distributed rather bilaterally at those early lags. That suggests that predictive structure building, as quantified by top-down node counts, poses strong demands on brain computation, in particular in early time windows. The fact that right-hemisphere activity contributed less to improving reconstruction accuracy might however indicate that these effects are less stable across participants. Another potentially informative observation in Figure 5 is that the left-corner TRF seems to combine aspects of both the top-down and the bottom-up effects. It peaks early, like the top-down TRF, but its time course is prolonged, which is the case for the bottom-up TRF as well. This observation is interesting because it aligns with the idea that the mildly predictive left-corner parsing strategy combines properties of top-down and bottom-up parsing." **(page 26, lines 557-573)**

We also updated the corresponding figure (Figure 2.3) and section in the revised Supplementary Information (Section S2.2; page 5, lines 63-67).

A related question, which is not completely described in the method section: how are the ROI results generated? Do you create a new average time-series per ROI per subject and then compute accuracies, or just average accuracies over the ROI's source points? How are the TRFs per ROI computed? Are the issues with polarity that I described above problematic here? There could be cancellations when averaging over ROIs, which could mask results. Rectifying TRFs could also be helpful here.

Response: To compute the reconstruction accuracy for a region of interest, we averaged the reconstruction accuracies of all source points in that region. TRFs per ROI were computed in the same way, by averaging the TRF in each individual source point. We have added a sentence to Section 3.3 (page 27, lines 592-594) explaining this.

In our initial submission, we averaged unrectified TRFs per ROI, but based on the Reviewer's comments we now present TRFs that were rectified before averaging across all ROI source points. Figures 6C and S2.4C were updated accordingly, and we also added pairwise comparisons between the response functions of the different syntactic features. We again thank the Reviewer for their suggestion to rectify TRFs before averaging, because the new TRF figures and results are more informative and interpretable in several ways: they better show the consistently bimodal response function of the top-down effect, they are more clearly linked to the reconstruction accuracy results (in Figures 6B and S2.4B, respectively), and they illustrate more clearly how the top-down and left-corner predictor are competing for explaining the same variance (Figures 6C versus 2.4C).
We have added the following discussion of the results to Section 3.3 of the revised manuscript:

"Cluster-based permutation tests (with alpha = 0.0056) were then used to compare the time courses of the syntactic predictors in each ROI. As shown in Figure 6C, the TRF of the top-down predictor has a higher amplitude than the TRFs of both the bottom-up and left-corner predictors in each ROI. In addition, the bottom-up response is stronger than the left-corner response in the IFG, corroborating the reconstruction accuracy results. What is also noteworthy is that the top-down TRF is bimodal in all ROIs, peaking at around 100 and 400 ms, and that the strongest peak of the bottom-up TRF falls in between those two peaks, around 300 ms, in the IFG and ATL." **(page 28, lines 603-609).**

We also updated the corresponding figure (Figure S2.4C) and section in the revised Supplementary Information (Section S2.3, page 6, lines 85-88).

Reviewer #3:

This paper has a very promising start, but it leaves the reader with an unclear outlook.

Major critical comments:

1. A first major point is that I am not sure whether the authors' goal is to uncover syntactic representations as they are deployed in time, or the processing issue of varying syntactic complexity. The metric is unclear to this end, even as originally proposed by Brennan. Therefore, it is unclear, at least to me, which cognitive processes the dependent measure taps into. I realize that the three parsing models tested are models of processing, but is the neural dependent measure a correlate of syntactic structure deployment (states), or of the incremental processes feeding into each state (changes across states)? A mixture of the two? It should be clarified.

Response: This is a very important question, and, in our view, not a question that anyone currently has an empirically supported answer to. We are ultimately interested in how syntactic representations incrementally emerge in neural tissue, but most experimental results that are used to address this topic are equally compatible with an interpretation in terms of (syntax-induced) processing cost. We therefore believe that it is currently not possible to distinguish between the representation-based and processing-based interpretations of our and related results in the literature.

To clarify our agnostic stance on this issue, we have reframed our introductory discussion in Section 1.1.1, where we introduce the different parsing models and possible interpretations of the resulting neural data:

"Note that our use of three different parsing methods does not imply that the human brain hosts multiple parsers. We use these methods as explicit and interpretable tools to derive values for incremental node count, a variable that counts the steps involved in syntactic structure building (e.g., the parser actions in Table 1). Thus, we use node counts as time-sensitive proxies for the operations that build structure in integratory or predictive ways, depending on how nodes (or, rather, parser actions) are counted. Neural responses associated with node count can therefore not be straightforwardly interpreted in terms of the representational properties of the syntactic structure being built. At present, we cannot distinguish between an interpretation of neural data in terms of evolving (syntactic) representations and an interpretation in terms of processing costs associated with incrementally deriving those representations. Thus, when node count derived from the top-down method significantly predicts brain activity, it does not mean that the resulting activity pattern reflects a partial, predictively activated syntactic representation, nor does it suggest that the brain is actually building hierarchical structure from the top of the tree to the bottom. Rather, it suggests that Dutch sentence comprehension can be characterized via a predictive mechanism, which postulates structure early in time." **(page 9, lines 181-194)**

2. A second major point is that syntactic information rate seems to be based on the assumption that not only syntactic complexity changes in time (numerator), but that the time range at the denominators is very much constrained, to a point that it makes sense to attribute any significant change in rate not to variability in the denominator, but rather to variability in the numerator. In the case at hand, this would mean that the rate of the specific medium - speech acoustics - is roughly constant, and what varies is the number of nosed closed (or opened) at each point in time. Such assumption does not stand on empirical grounds, but may have been inferentially derived from massive averaging approaches to speech acoustic analysis, which output a shallow energy peak in the low-theta, high-delta ranges. Those analyses are useful to approximate a range of interest, but the speech signal is much more dynamic on a local scale. And here is the key point: what counts as local scale in this paper? The authors use a very peculiar way of selecting relevant syntactic information (see point 6 below), sparsely extracting macroconstituents from the syntactic flow of information, a highly debatable choice which seems to artificially determine a roughly stable time variable in the denominator.

Response: We believe there has been a misunderstanding, and do not share the Reviewer's characterization of syntactic information rate for two reasons: First, as we use it, syntactic information rate does not rely on the assumption that "the rate of the specific medium – speech acoustics – is roughly constant". We therefore believe that it is informative about the temporal dynamics of syntactic information in speech, even if the speech rate turns out to be locally variable. Second, the variability in speech rate in our stimuli, while naturalistic, is actually rather constrained. We elaborate on both points below.

First, the Reviewer's assertion that local variability in speech rate interacts with our measure of syntactic information rate seems to come from misunderstanding about what information is used to compute syntactic information rate. In contrast to the Reviewer's characterization, syntactic information rate does not reflect the number of phrases (numerator) per unit of time (denominator). This interpretation of rate cannot apply to syntactic information, because phrases are not counted in the same dimension as time is. Because syntax is hierarchically structured, syntactic complexity reflects hierarchical embedding, which might be said to be counted in the vertical dimension. Units of time (and sequential order) instead are counted in the horizontal dimension. We therefore think that it is not accurate to define the rate of syntactic complexity as the number of phrases per time unit. Instead, syntactic information rate, as we use it, merely reflects the average time between two consecutive words that predict or integrate multiple phrases. This happens to be roughly between 0.25 and 2 seconds, hence the rate is 0.5-4 Hz. It is important to add that we do not claim that all syntactic variability in the signal will be covered by this frequency band. Rather, our argument is that the rate at which syntactic information is presented in these stories generally falls in the delta range (our "approximated range of interest"), which we therefore deem a syntactically informative timescale for the brain.

Second, the speech rate in our naturalistic stimuli was quite constrained. In order to compute the speech rate, we extracted the number of syllables in each story segment using the method described in de Jonge and Wempe (2009), which has been validated against manual annotations of Dutch. This method, which is implemented in Praat, automatically detects syllable nuclei based on intensity, pausing and voicedness, after which speech rate is expressed as the number of syllables per time unit. (Note that speech rate is computed differently from how we derived syntactic information rate. The reason that speech rate can

instead be expressed as the number of syllables per time unit is that, in contrast to phrases, syllables are sequentially concatenated in speech. They can therefore be counted in the same 'horizontal' dimension as time is.) First of all, this analysis showed that speech rate was very similar across the different stories (mean = 3.58 syllables/second, range across nine stories = 3.44-3.71). Importantly, the speech rate was also consistent within each story. The density plots below show the distribution of syllable duration (including median and quartiles) across all audiobooks. It can be seen that the variation in syllable duration is relatively constrained within each story (i.e., syllables are roughly regularly distributed in time; Poeppel & Assaneo, 2020), suggesting that, even on a local scale, the speech rate was not as variable as the Reviewer suggests.

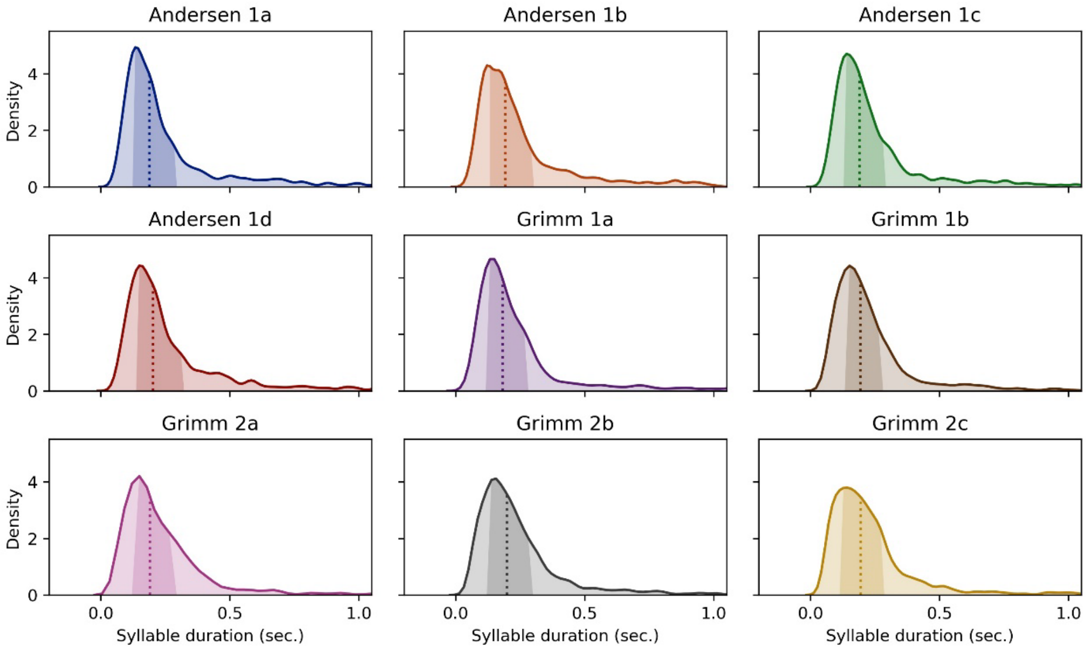

de Jong, N. H., & Wempe, T. (2009). Praat script to detect syllable nuclei and measure speech rate automatically. *Behavior Research Methods*, *41*(2), 385–390. https://doi.org/10.3758/BRM.41.2.385

Poeppel, D., & Assaneo, M. F. (2020). Speech rhythms and their neural foundations. *Nature Reviews Neuroscience*, *21*(6), Article 6. https://doi.org/10.1038/s41583-020-0304-4

3. A third major point is that the so-called "results linking syntactic processing to delta-band activity" is actually a set of theses or at best a mixed bag of results, including debated interpretations of frequency tagging findings and approaches using narrowband mutual information, the latter requiring aggressive low-pass filtering which almost invariably leads to false positives (MI is "created" by phase distortions due to LP filtering, rather than computed from the actual data). Hence, the motivation for the focus in the delta band does not have solid grounds. Which in turn greatly limits the outlook as well as the outcome of the paper.

Response: We understand the Reviewer's concern about the relationship between syntactic processing and delta-band activity. But we hope that the following three clarifications

alleviate some of their concern and skepticism. First, the (cor)relation between syntax and delta comes not only from frequency-tagging experiments or mutual-information analyses. Recent M/EEG experiments relying on other methodologies and analytic approaches have shown that manipulations of syntactic structure modulate delta-band activity as well. These effects show up in increased phase connectivity for (syntactically more complex) sentences compared to phrases (Bai et al., 2022), in increased delta(phase)-gamma(amplitude) coupling as a function of bottom-up node count (Weissbart & Martin, 2024), and in a relationship between (single-trial) delta phase and syntactic parsing choices (Henke & Meyer, 2021; Meyer et al., 2017; Meyer & Gumbert, 2018). The fact that these results were achieved with a variety of methods suggests that the relationship between syntactic processing and delta activity is not merely a by-product of experimental or methodological choices. Second, as we noted in our previous response, we do not claim that all syntactic variability in the signal will be captured by modulations in the delta band, nor do we argue that delta activity necessarily, uniquely, or always reflects syntactic processing. Rather, we suggest that neural activity in the delta band might carry information relevant for syntactic processing, and we think that this possibility is supported by empirical results (see also our revisions in response to the next comment). A third reason for why delta activity might be informative about syntactic processing is that delta activity is responsive to prosody (Bourguignon et al., 2013; Inbar et al., 2023; Rimmele et al., 2021), which is closely related to syntactic structure. Delta might therefore be responsive to syntax not only because it tracks syntactic structure, but also because it relies on prosodic modulations for the inference of syntactic structure. Delta modulations may also reflect other physiological consequences of representing syntax, namely the recruitment and coordination of distributed networks and their complex receptive fields.

To explore the relationship between prosody and syntax in delta-band activity, we conducted additional analyses with a prosodic predictor. These additional analyses are reported in the revised Supplementary Information S4 (pages 10-13) and discussed further in the revised manuscript in Section 4.1 (pages 32-33, lines 702-721). The prosodic predictor reflects the strength of prosodic boundaries at each word. It is positively correlated with bottom-up node count (r = 0.34) and left-corner node count (r = 0.19), but not with top-down node count (r = 0.04). This asymmetry is interesting, because it shows that the syntactic feature that explains most variance in neural activity (top-down) has no relationship to prosody. Conversely, the syntactic features that are positively correlated with prosody (bottom-up and left-corner) only weakly predict brain activity.

Importantly, prosodic boundary strength does not explain our syntactic effects. That is, when we explicitly controlled for prosody by including prosodic boundary strength as a regressor in our TRF models, the effects of all syntactic predictors remained present. The effect of top-down node counts was stable, but the variance explained by bottom-up and left-corner node counts was significantly reduced by the addition of prosodic boundary strength (see Figure S4.1), suggesting that these two syntactic features share neural variance with prosody. This relationship between prosodic boundaries and syntactic integration points to a potential role for prosody in supporting the perceptual inference of syntactic structure. Moreover, we believe that these results strengthen our arguments for looking at delta activity, which might track prosody to help some aspects of syntactic processing (specifically, syntactic integration) but not others (syntactic prediction).

Bai, F., Meyer, A. S., & Martin, A. E. (2022). Neural dynamics differentially encode phrases and sentences during spoken language comprehension. *PLOS Biology*, *20*(7), e3001713. https://doi.org/10.1371/journal.pbio.3001713

Bourguignon, M., Tiège, X. D., Beeck, M. O. de, Ligot, N., Paquier, P., Bogaert, P. V., Goldman, S., Hari, R., & Jousmäki, V. (2013). The pace of prosodic phrasing couples the listener's cortex to the reader's voice. *Human Brain Mapping*, *34*(2), 314–326. https://doi.org/10.1002/hbm.21442

Henke, L., & Meyer, L. (2021). Endogenous oscillations time-constrain linguistic segmentation: Cycling the garden path. *Cerebral Cortex*, *31*(9), 4289–4299. https://doi.org/10.1093/cercor/bhab086

Inbar, M., Genzer, S., Perry, A., Grossman, E., & Landau, A. N. (2023). Intonation Units in Spontaneous Speech Evoke a Neural Response. *Journal of Neuroscience*, *43*(48), 8189–8200. https://doi.org/10.1523/JNEUROSCI.0235-23.2023

Meyer, L., & Gumbert, M. (2018). Synchronization of Electrophysiological Responses with Speech Benefits Syntactic Information Processing. *Journal of Cognitive Neuroscience*, *30*(8), 1066–1074. https://doi.org/10.1162/jocn_a_01236

Meyer, L., Henry, M. J., Gaston, P., Schmuck, N., & Friederici, A. D. (2017). Linguistic bias modulates interpretation of speech via neural delta-band oscillations. *Cerebral Cortex*, *27*(9), 4293–4302. https://doi.org/10.1093/cercor/bhw228

Rimmele, J. M., Poeppel, D., & Ghitza, O. (2021). Acoustically driven cortical delta oscillations underpin prosodic chunking. *eNeuro*, *8*(4), 1–15. https://doi.org/10.1523/ENEURO.0562-20.2021

Weissbart, H., & Martin, A. E. (2024). *The Structure and Statistics of Language Jointly Shape Cross-frequency Neural Dynamics During Spoken Language Comprehension* (p. 2023.10.06.561087). bioRxiv. https://doi.org/10.1101/2023.10.06.561087

4. The variance in sentence duration (in number of words) is very high: lines 254-255, "791 sentences, which are on average 10.8 words long (range = 1-35, SD = 5.95)". That reflects variance in phrasal structure duration, so I am very doubtful that one single rate of syntactic processing can cover such variance, and I am even more convinced that it is an artefact of the macroconstituent selection approach.

Response: All longer sentences consist of clausal conjunctions (i.e., "..a.., *and* ..b..") and/or contain subordinate clauses (e.g., "*because* ..a.., ..b..", "*after* ..a.., ..b..", "*when* ..a.., ..b.."), which means that the average length of clauses is actually a lot lower than the average length of full sentences. By extension, the "variance in phrasal structure duration" is not as high as suggested. That being said, the variance in phrase duration is irrelevant for our conclusion about syntactic information rate, because the latter is not derived from, nor does it reflect, the length of phrases. Instead, we derive syntactic information rate per parsing strategy from the different node-count vectors, which contain no information about the duration of phrases (i.e., they do not encode when a phrase starts and when that same phrase ends). In fact, because phrases are hierarchically embedded in one another, phrase duration is extremely variable, and so there is no regular phrase rate in natural language (this point was made forcefully in a recent perspective article about syntax and oscillations by Kazanina & Tavano, 2023). But because syntactic information rate bears no relation to the duration of phrases, it is not necessary that "one single rate of syntactic processing can cover such variance [in phrase duration]".

To clear up any possible confusion, we have rewritten and expanded the following paragraph of Section 2.3, in which we clarify our intended interpretation of syntactic information rate:

"It has been suggested that the timescale of syntactic processing overlaps with the delta frequency range (Henke & Meyer, 2021; Meyer et al., 2020), which is consistent with the syntactic information rate in the nine story segments that were used in this study. Syntactic information rates were derived from the time between the onsets of consecutive words with node counts above 2. Because we aim to illustrate the rate at which syntactic structure building can be initiated in the stimuli, only syntactically demanding words (i.e., node count > 2) were included in the rate calculation. The probability density plots in Figure 3 show that the syntactic information rate for each parsing strategy in all story segments largely falls within the delta range. What this means is that the average time between two consecutive words that predict (top-down, left-corner) or integrate (bottom-up) multiple phrases is between 0.25 and 2 seconds. As such, the syntactic information rate provides information about the timescale at which syntax can be perceptually inferred from naturalistic speech. To be clear, it should not be understood as reflecting the average lengths of phrases, or the number of phrases per unit of time. Syntax builds hierarchical structures, so sentence structures cannot be described in terms of these sequential properties. Rather than being concatenated, phrases are hierarchically embedded in one another. The sequential length of phrases is therefore extremely variable, and there is no regular phrase rate in natural language (Coopmans et al., 2023; Kazanina & Tavano, 2023). We therefore think that it is not accurate to define the rate of syntactic complexity as the number of phrases per time unit. Here, we instead derive the syntactic information rate from the time between syntactically demanding words, as encoded in the different node-count vectors. It therefore reflects the general rate at which predictive or integratory structure building can be initiated. And because it bears no relation to the sequential lengths of phrases, our claim that the syntactic information rate falls in the delta range does not mean that each phrase can be covered by a delta cycle." **(page 16, lines 325-346)**

Kazanina, N., & Tavano, A. (2023). What neural oscillations can and cannot do for syntactic structure building. *Nature Reviews Neuroscience, 24*, 113–128. https://doi.org/10.1038/s41583-022-00659-5

5. I can understand the decision to leave out APs, but leaving out AdvP does not make much sense, given that they invariable include one or more, nested, NPs.

Response: We regret that it is unclear to us what the Reviewer means. First, adverbial phrases were not left out – all adverbs were analyzed as AdvP, which could modify verbs, adjectives, and other adverbs. And second, AdvPs never include noun phrases. If the Reviewer means complete phrases that function as adverbs (phrases like "on the last day"), these were analyzed like all other constructions, as transparent syntactic structures with hierarchical embedding (e.g., "on the last day" would be analyzed as a PP, consisting of an NP with a DP as specifier, an NP with an adjectival modifier, and so on).

6. The section at lines 300-306 reflects the key problem with this paper, in my opinion, that is the selection of what is relevant data for the determination of a syntactic information rate. First, did the authors count also lexical nodes or not? In other words, what is node = 1? Only by knowing that can one understand exactly what node = 2 really means as a threshold. Second, selecting such a

threshold is an arbitrary choice, bordering cherry picking (that is, selecting a data frame that yields results consistent with the unsubstantiated delta hypothesis). Are the authors only selecting macroconstituents for their syntactic analysis? And why would that be a meaningful way to go for a putative comprehension process? Do they assume that macroconstituents are processed in isolation by the rest of syntactic constituents by a separate sub-band of the delta band? I have a hard time with this choice of data analysis, honestly.

Response: Because every word has a categorial node, the minimal value of node count is 1. Thus, when node count = 1, only the syntactic category of the current word is counted (see Figure 2 in the manuscript). While we acknowledge that the chosen threshold of node count = 2 seems arbitrary, we think that it is a meaningful and syntactically justifiable threshold because many single words have a node count of 2 just by themselves. Computing the syntactic information rate at a threshold of node count = 1 will therefore result in a frequency very close to the word rate, which is not very informative. The reason that many single words have a node count of (at least) 2 just by themselves has to do with the fact that we adopted the X-bar template to annotate NPs and VPs. As we explain in the first paragraph of Section 2.3, we did this in order to make a structural distinction between complements and adjuncts, which are attached at different heights in the phrase (e.g., Carnie, 2021; Jackendoff, 1977). If these phrases did not have a complement, this meant that they had two unbranching nodes, which can be seen in Figure 2 at the nouns "moment", "vogel", and "kreet". The result is that these words have node count of at least 2, even if only the nodes belonging to that noun are counted.

To show that the estimation of syntactic information rate does not result from our "selection of what is relevant data", we computed a frequency decomposition of the original node count predictors. This analysis is completely unbiased because it uses all information present in the predictors (both timing and amplitude). The result, shown in the figure below, corroborates our custom computation of syntactic information rate. That is, it illustrates that, from an abstract syntactic perspective, there is relevant information in the (lower) delta range.

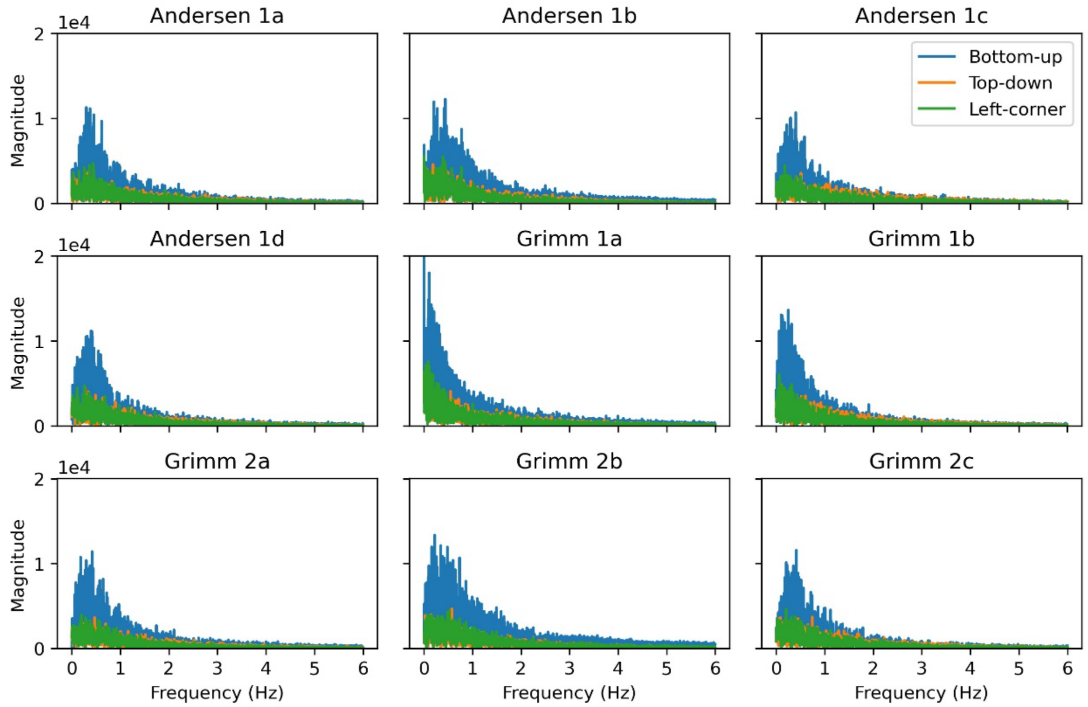

Last, we would like to add that we included this additional analysis and figure in the manuscript only to support our choice for the delta band. As we also said in response to the Reviewer's comment #3, we do not claim that there is anything inherently special about the delta band for syntactic processing. Rather, we suggest that activity in this frequency band can be informative about how syntactic processing takes place in the brain. This suggestion is based on the fact that previous studies have shown effects of syntactic processing in this band, and that certain aspects of syntactic variation in the stimulus – the temporal distribution of node count – reside in the delta timescale. This might be one of the reasons that previous studies have found syntactic effects in delta-band neural activity.

7. The Results section sees the top-down model largely outperforming the remaining two models. Given that only macroconstituents can enter the computation, is it really that surprising a finding? Can conclusions about syntax in general (see the Title and Discussion) be derived from arbitrarily selecting only some syntax of choice? At the very least, the authors should test the models on the whole syntactic flow of information.

Response: As we have explained in response to the Reviewer's previous comments about our use of syntactic information rate, it is not true that "only macroconstituents can enter the computation" or that we "arbitrarily selected only some syntax of choice". Instead, node count is computed for each individual word, and these word-based measures of syntactic complexity are regressed against continuously varying neural dynamics. In other words, our models are tested "on the whole syntactic flow of information".

8. Figure 6 is interesting but leaves the reader with the impression that a line of work could stem from there, but has been somewhat top-down interrupted.

Response: We have revised Figure 6, in which the analysis of the response functions in the different regions of interest is based on rectified TRFs. The new TRF figures, which now additionally include pairwise comparisons between the response functions of the different syntactic features, are more informative and interpretable in several ways: they show more clearly that the top-down effect is consistently bimodal across the three different ROIs (with the bottom-up TRF peaking in between the early and late top-down peaks in IFG and ATL), they are more clearly linked to the reconstruction accuracy results (in Figures 6B and S2.4B, respectively), and they illustrate more clearly how the top-down and left-corner predictor are competing for explaining the same variance (Figures 6C versus 2.4C).
We have added the following discussion of the results to Section 3.3 of the revised manuscript:

"Cluster-based permutation tests (with alpha = 0.0056) were then used to compare the time courses of the syntactic predictors in each ROI. As shown in Figure 6C, the TRF of the top-down predictor has a higher amplitude than the TRFs of both the bottom-up and left-corner predictors in each ROI. In addition, the bottom-up response is stronger than the left-corner response in the IFG, corroborating the reconstruction accuracy results. What is also noteworthy is that the top-down TRF is bimodal in all ROIs, peaking at around 100 and 400 ms, and that the strongest peak of the bottom-up TRF falls in between those two peaks, around 300 ms, in the IFG and ATL." **(page 28, lines 603-609).**

Minor critical comments:

1. Line 112: The reduction operation cannot be identical to the expansion operation (of which at line 107): VP > V NP. Please, revise.

Response: The Reviewer is correct that reduction and expansion are not the same operation. However, VP → V NP refers not to the operation but rather to the underlying rewrite rule, which reflects a relation between nodes and does not dictate any particular derivational procedure. It therefore does not distinguish between bottom-up and top-down parsing (see also Table 1). We have made this clear by adding "in the rewrite rule" to the first instance at which VP → V NP is mentioned (page 6, line 110).

2. Line 345: "We low-pass filtered the signal at 4 Hz using a zero-phase FIR filter (corresponding to the 0.5-4 Hz delta band)". Does this mean that the transition band is 1 Hz for both the 0.5 HP and the 4 Hz LP? If that is so, then the band that is actually untouched is 1-3.5 Hz. I would suggest considering either a longer filter with a smaller TB or LP at 4.5 Hz. The HP seems to be difficult to change, unless the authors consider a longer filter. MNE procedures generally do not pay sufficient attention to filtering issues.

Response: The band that is untouched is actually 0.5-4 Hz. For zero-phase FIR filters in MNE, the stop frequencies are computed as pass frequency +/- transition band, and the cutoff frequency, i.e. the -6 dB point, is in the middle of the transition band (see https://mne.tools/dev/generated/mne.filter.create_filter.html). For a FIR filter with MNE's default settings, the transition bands for 0.5 and 4 Hz are 0.5 and 2 Hz respectively, and the frequency response would therefore be as follows:

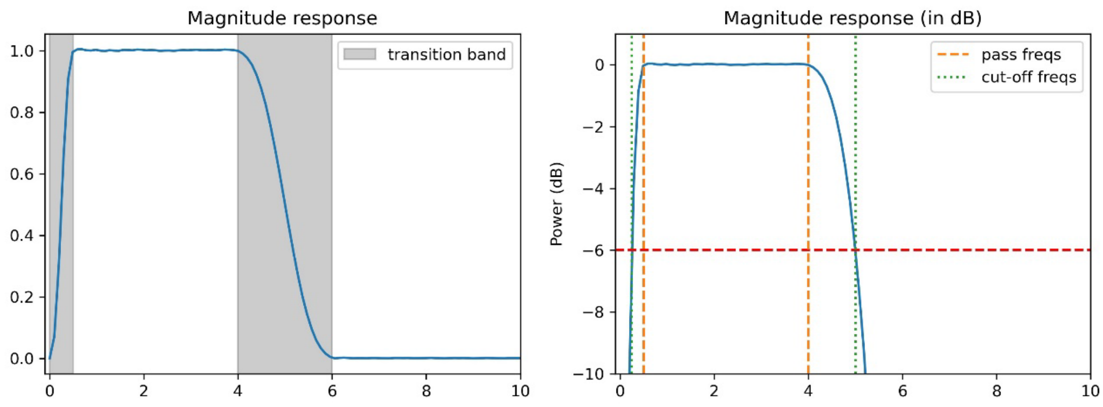

Major critical strengths:

1. I very much like the strategic use of the Dutch language mix headedness feature to test the validity of parsing strategies outside of the English language. I think this is the strong point of the paper. Had the authors then worked on the whole syntactic information flow rather than arbitrarily selecting some data to fit their hypothesis, it would have made for a nice paper.

2. I appreciate a lot Figure 3. I do not share the method that led to the figure, but I appreciate the clarity and thoroughness of the illustration.

Minor critical strengths:

1. I appreciate the use of the "Ffmpeg software" instead of RMS for loudness normalization. However, was is the loudness level chosen to be equal for all participants or based on an individually measured threshold?

Response: The loudness level was equal for all participants. We have added this information to the revised manuscript in Section 2.2.

In conclusion, I appreciate the technical effort, and commend the authors on the good quality of the illustrations, but I cannot get on board with the data selection, as it seems not to be data-driven, or even hypothesis-driven, as it is very hard to hypothesize that somewhat complex syntax lives in a frequency of its own. I am sorry I cannot be more positive.
